# COVID-19 modeling and non-pharmaceutical interventions in an outpatient dialysis unit

**DOI:** 10.1371/journal.pcbi.1009177

**Published:** 2021-07-08

**Authors:** Hankyu Jang, Philip M. Polgreen, Alberto M. Segre, Sriram V. Pemmaraju

**Affiliations:** 1 Department of Computer Science, The University of Iowa, Iowa City, Iowa, United States of America; 2 Department of Internal Medicine, Division of Infectious Diseases, Carver College of Medicine, The University of Iowa, Iowa City, Iowa, United States of America; University of Zurich, SWITZERLAND

## Abstract

This paper describes a data-driven simulation study that explores the relative impact of several low-cost and practical non-pharmaceutical interventions on the spread of COVID-19 in an outpatient hospital dialysis unit. The interventions considered include: (*i*) voluntary self-isolation of healthcare personnel (HCPs) with symptoms; (*ii*) a program of active syndromic surveillance and compulsory isolation of HCPs; (*iii*) the use of masks or respirators by patients and HCPs; (*iv*) improved social distancing among HCPs; (*v*) increased physical separation of dialysis stations; and (*vi*) patient isolation combined with preemptive isolation of exposed HCPs. Our simulations show that under conditions that existed prior to the COVID-19 outbreak, extremely high rates of COVID-19 infection can result in a dialysis unit. In simulations under worst-case modeling assumptions, a combination of relatively inexpensive interventions such as requiring surgical masks for everyone, encouraging social distancing between healthcare professionals (HCPs), slightly increasing the physical distance between dialysis stations, and—once the first symptomatic patient is detected—isolating that patient, replacing the HCP having had the most exposure to that patient, and relatively short-term use of N95 respirators by other HCPs can lead to a substantial reduction in both the attack rate and the likelihood of any spread beyond patient zero. For example, in a scenario with *R*_0_ = 3.0, 60% presymptomatic viral shedding, and a dialysis patient being the infection source, the attack rate falls from 87.8% at baseline to 34.6% with this intervention bundle. Furthermore, the likelihood of having no additional infections increases from 6.2% at baseline to 32.4% with this intervention bundle.

## Author summary

As we write this, the COVID-19 pandemic has essentially taken over the world, with more than 20 million cases spread over 216 countries. A big concern for policy makers all across the world has been the impact of COVID-19 on healthcare systems and whether these systems can cope with the enormous strain placed on them by COVID-19. In this paper, we consider the spread of COVID-19 in a specific healthcare setting: the outpatient dialysis unit. Hemodialysis patients are extremely vulnerable to infections in large part due to multiple immune-system deficiencies associated with renal failure and hemodialysis. Hemodialysis facilities also increase the risk of COVID-19 transmission because each patient is in frequent, close contact with other patients and healthcare personnel. Thus, a dialysis unit can be seen as a microcosm for the worst-case impacts of COVID-19 in a healthcare setting. In this manuscript, we show via high-fidelity modeling and simulations that under pessimistic modeling assumptions, there is a combination of relatively simple, inexpensive, and practical non-pharmaceutical interventions that can substantially lower the impact of COVID-19 in the dialysis unit. Our simulations are based on fine-grained healthcare personnel movement data that we make available for other modelers to use.

## Introduction

As of August 13, 2020, 5.2M people in the United States were infected with the COVID-19 virus, with more than 166K of these cases resulting in death of the patient [1]. Using data from 13 US states, the CDC estimates that among adults hospitalized due to COVID-19 during March 1–May 31, 2020, 5.9% were HCPs and of these, nursing-related occupations (36.3%) represented the largest proportion [2] The CDC reports that 132K US healthcare personnel (HCPs) were infected as of August 13, though this is likely a substantial underestimate because information on whether an individual was an HCP was available for only 22.2% of the total cases. Thus healthcare settings represent a significant locus of COVID-19 transmission for HCPs.

Infections are the second leading cause of mortality among hemodialysis patients [3], and rates of sepsis are approximately 100 times greater than in the general population [4]. Almost 20% of patients on hemodialysis develop pneumonia in their first year [5], and rates of pneumonia among dialysis patients are three to five times higher than in the general population [6, 7]. Risk of infections and adverse outcomes attributable to infections are in large part due to multiple immune-system deficiencies associated with renal failure and hemodialysis [8–10].

Patients undergoing hemodialysis are especially at risk for acquiring COVID-19, and to date, several COVID-19 outbreaks have occurred in dialysis centers in multiple countries [11–15]. Hemodialysis facilities may increase the risk of COVID-19 transmission and other respiratory droplet infections because each patient is in frequent, close contact with other patients and HCPs: most dialysis centers are large spaces without separation between patients [16]. The efficiency of transmission in this setting is also increased by the fact that a small team of HCPs usually attends to a larger set of patients, and that dialysis unit HCPs are difficult to replace due to their specialized skills. Furthermore, patients undergoing dialysis experience additional healthcare exposures beyond their usual hemodialysis center. For example, patients on dialysis are frequently hospitalized and often reside in nursing homes [3, 17, 18]. In addition to acquiring COVID-19, dialysis patients may be at greater risk of developing more severe cases as they often suffer from many chronic diseases, e.g., diabetes and hypertension, that have been linked to greater COVID-19 morbidity and mortality [19].

This paper explores the effectiveness of simple, practical, and low-cost non-pharmaceutical interventions (NPIs) in reducing the spread of COVID-19 in one specific healthcare setting: the outpatient dialysis unit. Our work uses computational modeling and discrete-event simulations to evaluate the effectiveness of six specific simple, low-cost NPIs in reducing the spread of COVID-19 in a dialysis unit. Our high-fidelity simulations are based on fine-grained HCP movement and interaction data collected in an outpatient hospital dialysis unit at the University of Iowa Hospitals and Clinics (UIHC). We have used this data in previous work, wherein we have simulated the spread of Methicillin-resistant *Staphylococcus aureus* (MRSA) in a dialysis unit and investigated the effectiveness of interventions such as architectural changes to the unit [20]. In this work, we test the candidate NPIs both alone and in combination, using a set of disease models that represent alternate possibilities of how COVID-19 is transmitted and unfolds. The NPIs under study are: (*i*) voluntary self-isolation; (*ii*) active syndromic surveillance and compulsory isolation; (*iii*) the use of masks (HCPs, patients) and/or N95 respirators (HCPs); (*iv*) improved social distancing among HCPs; (*v*) increased physical separation between dialysis stations; and (*vi*) patient isolation combined with the preemptive isolation of exposed HCPs. A description of these NPIs appears in the section Methods: NPIs.

As we evaluate these NPIs, we focus not just on their effect on COVID-19 spread, but also on the cost of implementing these NPIs. For the NPIs we consider, cost can be measured by the number or HCPs temporarily replaced and the duration of this replacement, the number of N95 respirators used and the duration of this usage, and the number of dialysis patients sent to isolation unit. The main takeaway from our work is that even though under pre-COVID-19 usage conditions COVID-19 can have near-100% attack rate in the dialysis unit within 30 days of the first agent becoming infected, it is possible to design and implement a low-cost NPI bundle that leads to a substantial reduction in the attack rate and likelihood of COVID-19 spread in the dialysis unit.

## Methods

### Ethics statement

We did not gather any patient data from our instrumentation of the dialysis unit. All HCP movement that we gathered was completely anonymous. Badges were arbitrarily distributed to HCPs at the start of each day, ensuring that there was no necessary connection between HCPs with the same badge ID across different days. For all these reasons, this project was human subjects research exempt.

The UIHC dialysis unit serves patients six days a week. Patients typically dialyze three times a week either Monday, Wednesday, and Friday (MWF) or Tuesday, Thursday, and Saturday (TThS)(either MWF or TThS) in one of three daily blocks (morning, afternoon, or evening). Interactions between patients and HCPs form an implicit underlying *contact network* which, along with an explicit model of how the infection is transmitted between agents, form the basis for agent-based simulation. A typical simulation study, then, introduces a single infected agent into a virtual dialysis unit defined by the contact network, the disease model, and the particular set of NPIs being tested. Outcomes from each trial, typically in the form of total number of infected individuals and the cost of the NPI, can be aggregated over multiple replicates having the same experimental conditions and then compared across conditions to determine the effectiveness of the NPIs under study.

### Temporal contact networks

**Fig 1:**
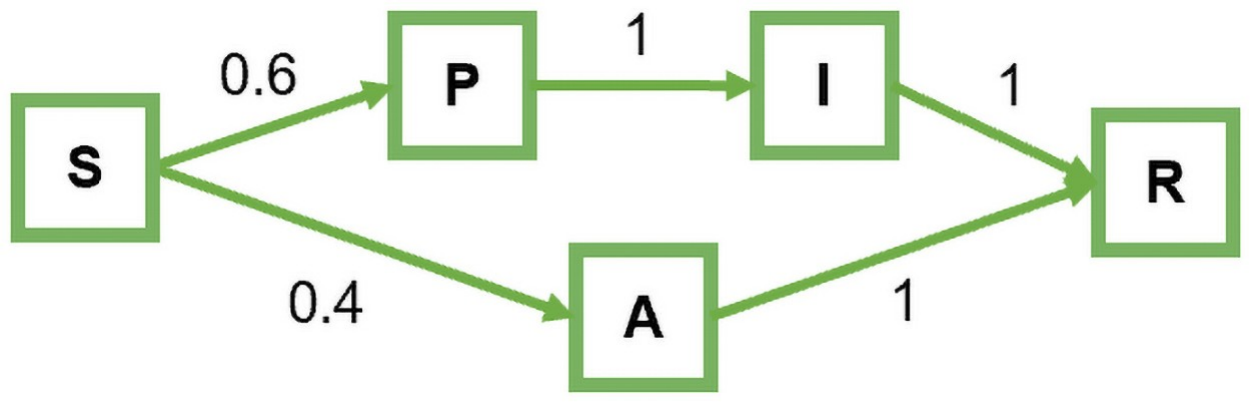
Agent state transition graph. Agents are always in one of five mutually exclusive states: susceptible (*S*), presymptomatic (*P*), infected with symptoms (*I*), asymptomatically infected (*A*), or recovered (*R*).

As described in [20], ten days of deidentified HCP movement and interaction data were gathered at a dialysis facility at the UIHC in the fall of 2013 [21]. Twenty two line powered *beacons* were placed in static locations including at each dialysis station, and rechargeable *badges* were distributed to HCPs in the unit. There are 9 dialysis chairs placed along the perimeter of a rectangular room, a nurses’ station in the center, a bathroom and two handwashing stations. For a schematic of the dialysis unit, including locations of the beacons, consult our previous work [20] (Fig 1). Each badge broadcasts its identity every few seconds, while beacons record and timestamp each message received, along with an indication of its received signal strength index (RSSI). RSSI increases with proximity, a fact later used to reconstruct the location and movement of HCPs within the unit. Because patients were not badged, dialysis sessions were imputed based on knowledge of the unit’s usual patient schedule and the observation that a HCP typically spends a prolonged time at a dialysis chair at the start and end of each dialysis session [20, 22]. We cast this problem as a binary classification problem whose aim is to classify, for each chair, every time window as either the start/end of a dialysis session in that chair or not. This classification is based on the distance profiles of all HCPs to with respect to a chair during a time window. To implement this idea, we precompute the distances of all HCPs to all the dialysis chairs over the observed timesteps. Then, for each chair, we manually select HCP distance profiles that correspond to long-duration, close contacts and mark these as positive instances. We also manually select distance profiles that are not long-duration close contacts and mark these as negative instances. We then train a binary classifier on these positive and negative instances. Finally, for each dialysis chair and every time window, we apply the trained classifier to classify the distance profiles of HCPs to that chair during that time window. This process identifies, for every dialysis chair some time windows as the start/end times of patient dialysis sessions. This process is described in more detail in [20]. Moreover, because data were collected anonymously thereby precluding linkage across multiple days, our simulations rely on repeating a single day’s data to create a 30 day simulation.

Our system for imputing dialysis sessions detected between 13 and 20 patient sessions over 6 deployment days [20]. To simulate a worst-case scenario, we picked the day with 20 dialysis sessions to repeat. Thus 20 patients had dialysis sessions on a MWF schedule and another 20 on a TThS schedule. There were 11 HCPs present in the data on this day. We generated 30 days of simulated unit operations by randomly assigning patients to dialysis stations on a consistent (morning, afternoon, or evening) block schedule. This results in 6–7 dialyzing patients per block, with a total population of 51 agents (40 patients and 11 HCPs) in the simulation. By replaying the HCP trajectories in parallel with the imputed patient dialysis sessions, we reconstructed all contacts (where a *contact* is defined as two agents, either HCPs and/or patients, within 6 feet of each other) between pairs of agents at 8 second intervals.

From these contact data, we generated undirected, unweighted, temporal contact networks *G*^*d*,*t*^ = (*V*^*d*,*t*^, *E*^*d*,*t*^) where *d*, 0 ≤ *d* ≤ 29, represents a day in simulation, and *t*, 0 ≤ *t* ≤ 6821, represents each 8 second timestep within a roughly 15 hour day starting at 5:31 AM. Here *V*^*d*,*t*^ denotes the set of nodes (patients and HCPs) that participate in a contact on day *d*, at timestep *t* and *E*^*d*,*t*^ consists of all edges {*x*, *y*}, representing pairwise contacts between agent *x* and agent *y* occurring on day *d*, at timestep *t*. Note that the graphs *G*^*d*,*t*^ and *G*^*d*′,*t*^, for *d* ≠ *d*′, are quite similar because agent-agent contacts are obtained by replaying identical HCP movement patterns every day. However, there are differences between these graphs that are induced by differences in start/end times of dialysis sessions and assignment of patients to dialysis chairs. Let *V* = ∪_*d*,*t*_ *V*^*d*,*t*^ be the collection of patients and HCPs over the course of the entire simulation. Let *V*_*H*_ and *V*_*P*_ = *V*∖*V*_*H*_ denote the set of HCP nodes and patient nodes, respectively; |*V*_*H*_| = 11 and |*V*_*P*_| = 40. Let *E* = ∪_*d*,*t*_ *E*^*d*,*t*^ denote the set of contacts throughout the simulation period. To each edge {*x*, *y*} in *E* we assign a positive integer weight that is the number of times the contact between agent *x* and agent *y* occurred over all the temporal graphs *G*^*d*,*t*^. Finally, let *G* = (*V*, *E*) denote the static, undirected, weighted, contact network of all HCPs and patients.

### Shedding models

Fig 1 shows a probabilistic state transition diagram of an individual agent’s disease states. At each timestep of the simulation, a susceptible agent (in state *S*) will either become infected or remain susceptible, depending on their interactions with other agents and in accordance with the shedding model (see below). Probabilistically, 60% of the agents who become infected do so with symptoms and transition into the presymptomatic state *P* [23–27]. The remaining 40% become asymptomatically infected and transition into state *A*. The time, *period*_*P*_, that an agent spends in state *P* is geometrically distributed with an expected length of 6 days [23, 28–31]. Following state *P*, an agent spends *period*_*I*_ days—geometrically distributed with expected length of 7 days—in the symptomatically infected state *I* [32–36]. Symptoms emerge in agents immediately after *period*_*P*_ days, i.e., as they transition from state *P* to state *I*. Agents who enter state *A* stay in that state for a total of *period*_*P*_ + *period*_*I*_ days. In other words, we assume that there is no difference in the total period of infection between patients who are symptomatic and asymptomatic patients. On leaving state *I* or *A*, agents recover and become immune to reinfection. This is represented by state *R*.

**Fig 2:**
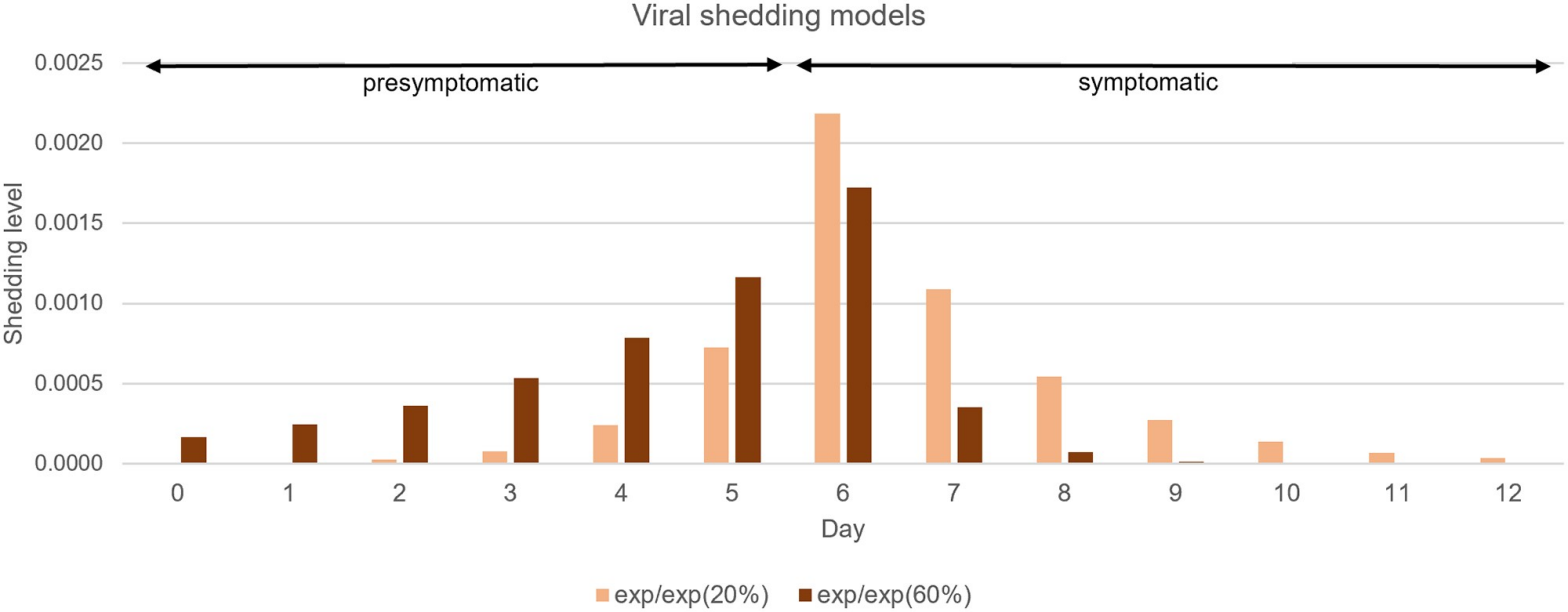
Viral shedding models. The bar charts show the shedding level of an infected individual whose presymptomatic period and symptomatic period are 6 and 7 days, respectively. The percentage in the parenthesis of each model corresponds to the percentage of shedding during the presymptomatic state (*P*) relative to the total shedding volume. The peak shedding level differs in the two models, but the total shedding volume remains the same.

In this study, we consider a viral shedding model with an exponential ramp up followed by an exponential ramp down in daily shedding level (see Fig 2). Specifically, we use the notation *exp/exp (f%)* to denote a viral shedding model with the following properties:

(i) The shedding level for every COVID-19 cases, whether symptomatic or not, exponentially ramps up daily, reaches a maximum, and then exponentially ramps down daily.
(ii) The length of the ramp up period is assumed to be exactly *period*_*P*_ and the length of the ramp down period is assumed to be exactly *period*_*I*_. This implies that for a symptomatic COVID-19 case, the day of maximum viral shedding is the first day in which symptoms emerge.
(iii) The exponential ramp up and ramp down factors are chosen so that *f*% of the total shedding occurs in the presymptomatic state.

The ramp up and ramp down aspects of this model and the timing of the peak viral shedding are inspired by experimental evidence for similar behavior in influenza and SARS, where viral loads initially build, peak at onset of symptoms, and decline thereafter [36–38].

Different instantiations of this model are obtained by using different values of *f*. In this paper, we show results for *f* = 20 and *f* = 60 (see Fig 2). These values of *f* yield the percentage of transmission occurring prior to symptom onset in the 30%—70% range [23, 36, 39]. The proportion of presymptomatic viral shedding *f* is somewhat lower than the proportion of presymptomatic transmission because there remain fewer susceptible agents in the latter days of the simulation. In Section “Calibrating the shedding model”, we describe our shedding model more precisely, explain how it is parameterized, and describe a method to solve for model parameters so that the model is appropriately calibrated.

We assume that the infectiousness of an individual who ends up in state *A* is 75% of the infectiousness of an individual who is symptomatically infected over the course of their illnessWe assume that the 40% of individuals who end up in state *A* are uniformly 75% less infectious than those in state symptomatically infected over the course of their illness, reflecting what some have found to be reduced viral loads and a concomitant reduction in viral shedding on the part of asymptomatic agents [23–26, 38, 40, 41].

### Calibrating the shedding model

Our shedding model needs to be calibrated so that three constraints are met. First, we require that the shedding during the presymptomatic period be a fraction *f* of the total shedding. Second, we require that the resulting simulation yields a given target *R*_0_ in expectation in the scenario where the initial infected is a dialysis patient in the morning session of the first day. We use 2, 2.5, and 3 as target values for *R*_0_ and this is in line with estimated *R*_0_ values for COVID-19 [23, 31, 42, 43]. Third, for two alternate shedding models *exp/exp (f*_1_*%)* and *exp/exp (f*_2_*%)* with the same target *R*_0_, we also require that the total shedding volume for both models be identical. For example, for a particular target *R*_0_ value, (e.g., *R*_0_ = 3), we require that the *exp/exp (20%)* and *exp/exp (60%)* models both have the same total shedding volume, though this is achieved via different “shapes,” as can be seen in Fig 2. This last requirement is to ensure that there are no subtle differences in attack rates across the two alternate models that are caused by differences in total shedding volume.

Let *s*_*d*_ denote the *shedding level* of an agent *u* on day *d* of that agent’s infection. Without loss of generality, we assume that the maximum value of *s*_*d*_ is 1 unit. Let the shedding ramp up parameter be denoted *β* > 1 and the shedding ramp down parameter be *γ* > 1. This implies that the shedding levels are given by the sequence

$$\begin{array}{c} \ldots \frac{1}{\beta^{2}},\frac{1}{\beta },1,\frac{1}{\gamma },\frac{1}{\gamma^{2}},\ldots \end{array}$$

For a given value of *f*, the parameters *β* and *γ* are related by the equation

$$\begin{array}{c} (1-\frac{f}{100})\cdot (\sum_{i=1}^{6}\frac{1}{\beta^{i}})=\frac{f}{100}\cdot (\sum_{j=0}^{6}\frac{1}{\gamma^{j}}). \end{array}$$ (1)

As a result, for a given *f*, we can view *s*_*d*_ as being determined by a single parameter *γ*.

Now consider a contact between an infected agent *u* and a susceptible agent *v* on day *d* of *u*’s infection. The probability *p*_*d*_ of *u* transmitting infection to *v* via this contact is proportional to *u*’s shedding level *s*_*d*_ on that day, i.e.,

$$\begin{array}{c} p_{d}=\alpha \cdot s_{d} \end{array}$$ (2)

for a scaling parameter *α*. Since *s*_*d*_ = 1 on the day *d* at which the shedding level is maximum (which is also the day on which symptoms emerge), the parameter *α* has the interpretation that it is the probability of transmitting infection via a single contact on the day on which symptoms first emerge. Let *L* = *period*_*E*_ + *period*_*I*_ − 1 denote the index of the last day that an individual is shedding. Let *x*_*d*_(*u*, *v*) denote the number of contacts *u* has with *v* on day *d*. For each contact, the disease is transmitted with the probability of *p*_*d*_. Therefore, the event that *u* fails to infect *v* on day *d* can be expressed as a conjunction of independent events in which *u* fails to infect *v* in each contact. Letting $f_{d}^{u,v}$ denote the probability that *u* fails to infect *v* on day *d*, we get:

$$\begin{array}{c} f_{d}^{u,v}=(1-p_{d}{)}^{x_{d}\left(u,v\right)} \end{array}$$ (3)

Let *P*_*u*,*v*_ denote the probability that *u* infects *v* at some point in the simulation; this can be computed by 1 minus the probability that *u* fails to infect *v* from day 0 to day *L*:

$$\begin{array}{c} P_{u,v}=1-f_{0}^{u,v}\cdot f_{1}^{u,v}\cdots f_{L}^{u,v} \end{array}$$ (4)

**Fig 3:**
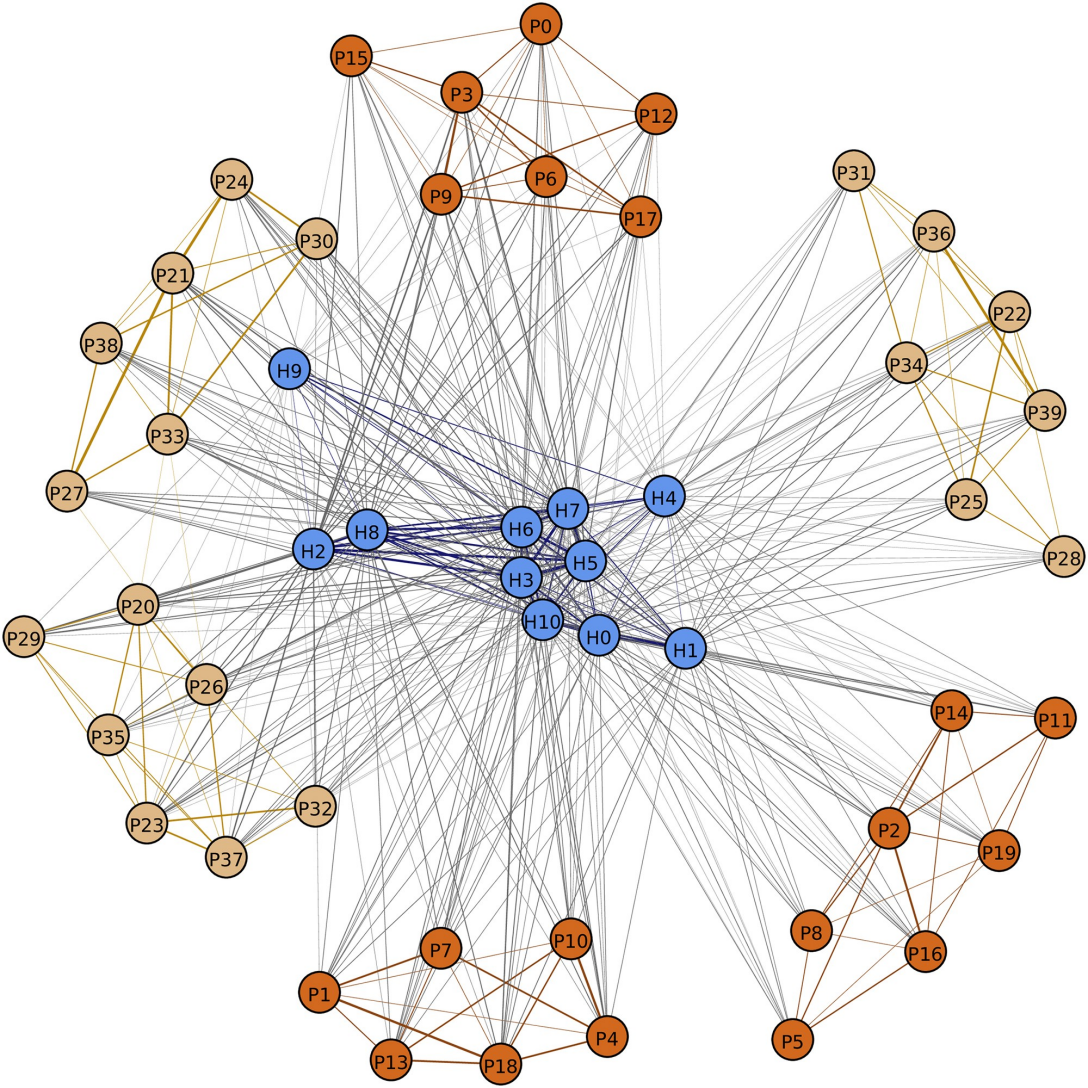
Contact network *G*. HCP nodes, MWF patient nodes, and TThS patient nodes are depicted in blue, chocolate, and burlywood colors, respectively. Contacts within the same type of nodes are represented as edges colored according to node type. Grey colored edges represent HCP-patient contacts. The thickness of the edge corresponds to the contact duration. Patients mostly have contact with other patients at the same dialysis session unless there is an overlap between sessions, hence we observe 6 patient clusters (3 sessions per day on MWF and TThS) of 6 or 7 patients each. For any session, a patient will be in contact with at most 2 neighboring patients, thanks to how neighboring dialysis chairs are positioned. Over several sessions, depending on which chair they occupy, a patient may come in contact with several other patients.

At this point, we have computed the probability of an infectious agent *u* infecting a susceptible agent *v*. Now referring to Fig 3, let $V_{P}^{\prime }\subseteq V_{P}$ denote the set {*P*_0_, *P*_3_, *P*_6_, *P*_9_, *P*_12_, *P*_15_, *P*_17_} of patients who undergo dialysis in the morning on the first day of the simulation.

Note that the infection source is randomly chosen from $V_{P}^{\prime }$ and the rest of the agents are susceptible, which allows the use of linearity of expectation to compute *R*_0_ of the simulation:

$$\begin{array}{c} R_{0}=\frac{1}{|V_{P}^{\prime }|}\sum_{u\in V_{P}^{\prime }}\sum_{v\in V_{H}\cup V_{P}\setminus \left\{u\right\}}P_{u,v} \end{array}$$ (5)

Recall that this is the *R*_0_ for the scenario in which the infection source is a dialysis patient who arrives for dialysis in the morning session of the first day.

In Eq (5), *R*_0_ is a function of (*i*) the number of contacts *x*_*d*_(*u*, *v*) between agents *u* and *v* on a day *d*, as specified by the contact network, (*ii*) the daily shedding level *s*_*d*_ of an agent, and (*iii*) the scaling parameter *α*. Since the contact network is given and we have a target value of *f*, via Eq (5) we see that *R*_0_ is a function of parameters *α* and *γ*.

For the final stage of our model calibration, let *T* denote the total volume of shedding of infectious individual. This volume can be expressed in terms of the parameter *γ* as follows.

$$\begin{array}{c} T=\alpha \cdot (\frac{1}{1-\frac{f}{100}})\cdot (\sum_{j=0}^{6}\frac{1}{\gamma^{j}}) \end{array}$$ (6)

Eqs 1, 5 and 6 express the three constraints our calibrated shedding models needs to satisfy. We use these equations to compute parameter values for our models as follows. Suppose that we have a target *R*_0_ and two target shedding models *exp/exp (f*_1_ *%)* and *exp/exp (f*_2_ *%)*.

To solve for the parameters of the model *exp/exp (f*_1_ *%)*, we pick an arbitrary value for parameter *γ*, denoted *γ*_1_. This fixes the value *β*_1_ for parameter *β* as per Eq 1 and completely defines the shedding level *s*_*d*_ for all days *d*. We then use Eq 5 to solve for a value *α*_1_ for parameter *α*. Since *R*_0_ grows monotonically with *α*, to find an appropriate value of *α* that yields a target *R*_0_, we simply use binary search over the space of *α*. The triple (*α*_1_, *β*_1_, *γ*_1_) provides a complete calibration of the shedding model *exp/exp (f*_1_ *%)*, yielding the target *R*_0_.

We next solve for a triple (*α*_2_, *β*_2_, *γ*_2_) to calibrate model *exp/exp (f*_2_ *%)* as follows. Since we want to preserve total shedding volume across shedding models *exp/exp (f*_1_ *%)* and *exp/exp (f*_2_ *%)*, we use the total shedding volume for the shedding model *exp/exp (f*_1_ *%)* as the left hand side of Eq 6. This equation, along with Eq 5, constitute a system of two non-linear equations defined on variables *α* and *γ*. We solve for values *α*_2_ and *γ*_2_ using an iterative grid search which is aided by the fact that *R*_0_ is monotonic in *α* (see Eq 5) and *T* is monotonic in both *α* and *γ* (see Eq 6). Given *f*_2_, we can use Eq 1 to compute the value *β*_2_ of parameter *β* from *γ*_2_.

Using the above method, for *R*_0_ = 3, we obtain values *β* = 3.01 and *γ* = 2.0 for the *exp/exp (20%)* shedding model and *β* = 1.479 and *γ* = 4.90 for the *exp/exp (60%)* model. These two shedding models are illustrated in Fig 2. The value of *α* computed for these two models is different, as reflected by the different peak shedding levels in the two models. However, the total shedding volume is the same (by design). The calibrated values of the parameters for the two shedding model are reported in Table 1.

**Table 1:** Calibrated values of the parameters for the shedding model.

	*exp/exp (20%)*			*exp/exp (60%)*		
*R*_0_	*α*	*β*	*γ*	*α*	*β*	*γ*
2.0	1.29e-3	3.01	2	1.009e-3	1.475	4.65
2.5	1.712e-3	3.01	2	1.355e-3	1.479	4.85
3.0	2.183e-3	3.01	2	1.723e-3	1.479	4.9

### NPIs

We used our simulator to explore the impact of six different NPIs, both individually as well as in certain plausible combinations. These six NPIs are described in the next six subsections.

#### Voluntary self-isolation

Voluntary self-isolation refers to the practice wherein a symptomatic HCP elects to stay home for 14 days starting the day after symptoms begin with voluntary isolation compliance rate *r*_*VI*_ ∈ {0.5, 0.7}. While in isolation, the HCP is replaced by a temporary HCP, selected from a community with COVID-19 attack rate of 0.347% (the COVID-19 attack rate in Johnson County, IA as of early-August 2020, computed by dividing active cases by the population) [44]. If the incoming replacement is exposed to COVID-19, we make the worst-case assumption that the substitute is in the first day of their presymptomatic period.

#### Active syndromic surveillance and compulsory isolation

Active syndromic surveillance refers to the practice of testing HCPs before their shift, and sending all HCPs with symptoms home. As with voluntary self-isolation, the isolation period is 14 days, and the HCP is replaced with a temporary HCP selected from the community. Unlike voluntary self-isolation, however, isolation in this case is not optional, so the compliance rate is by definition always 100%.

#### Using masks and/or N95 respirators

Surgical masks and N95 respirators interfere with the ability of an infected agent to transmit the virus to a susceptible agent. The difference between the two is the ability to attenuate transmission: our model attenuation factors are 0.93 (for N95 respirators) and 0.4 (for surgical masks) in accordance with results found in the literature [45, 46]. In this paper, we consider three plausible mask interventions: (*i*) patients wear surgical masks and HCPs are unmasked; (*ii*) both patients and HCPs wear surgical masks; and (*iii*) patients wear surgical masks and HCPs wear N95 respirators. We assume that all agents are 100% compliant, all the time.

#### Improved social distancing among HCPs

Limiting physical interactions is an effective intervention for reducing the transmission of COVID-19 [47]. Social distancing in the dialysis unit refers to the practice of HCPs staying at least 6 feet away from each other when not directly engaged in patient care. In our simulations, we model this form of social distancing by removing a given fraction *r*_*SD*_ ∈ {0.25, 0.5, 0.75, 1} of contacts that occur between pairs of HCPs when outside the immediate proximity of a dialysis station.

#### Increased physical separation between dialysis stations

Dialysis chairs are placed roughly 6 feet apart from each other in the UIHC dialysis unit, just at the outer limit of the generally accepted respiratory droplet range. As a result, infected patients may be close enough to infect patients in adjacent chairs, and, more to the point, HCPs attending to patients in adjacent chairs may be quite close to each other. In our simulations, we model increased physical separation between dialysis stations by removing a given fraction *r*_*PS*_ ∈ {0.25, 0.5, 0.75, 1} of contacts between patients at adjacent dialysis chairs that are within 6 feet distance, and between HCPs that had contacts at adjacent dialysis stations.

#### Patient isolation and preemptive isolation of exposed HCPs

Many dialysis centers, like the UIHC, have an isolation room, where infectious patients may undergo dialysis without endangering others. For this intervention, we assume the first symptomatic patient in the unit will henceforth receive dialysis treatment in the isolation room. Preemptive isolation of exposed HCPs refers to the practice of placing the top *k* HCPs (*k* ∈ {1, ⋯, 5}) having had the most exposure to that now isolated symptomatic patient in compulsory isolation, replacing them with temporary HCP selected from the community. Unlike active syndromic surveillance and compulsory isolation, isolation is being imposed here based on the HCPs likely increased risk of infection as opposed to any actual evidence of infection. For small values of *k* (e.g., *k* = 1, 2), it is possible to implement this intervention simply by assigning primary and/or secondary HCPs to each dialysis session with the understanding that the primary HCP will provide most care during the session and will be assisted by the secondary HCP as needed.

#### NPIs in combination

We hypothesize that combinations of low-cost versions of these interventions could more effectively reduce the attack rate: we therefore explore the following plausible combinations of interventions:

- Baseline+: Surgical masks for all HCPs and patients, improved social distancing among HCPs (*r*_*SD*_ = 0.25), and increased physical separation between dialysis stations (*r*_*PS*_ = 0.75).
- Baseline++: All Baseline+ interventions plus patient isolation and preemptive isolation of exposed HCPs (*k* = 1) upon detection of the first symptomatic patient.
- Baseline+++: All Baseline++ interventions plus use of N95 respirators by all HCPs for 2 weeks following detection of the first symptomatic patient.

Note that the extra interventions included in Baseline++ and Baseline+++ are triggered by the detection of the first symptomatic patient. This approach is an attempt to keep interventions targeted and their costs low.

### Simulation setup

We create 6 models by independently using three different values of *R*_0_ ∈ {2.0, 2.5, 3.0} and two viral shedding models *exp/exp (20%)* and *exp/exp (60%)*. In addition, we consider the following two initial conditions.

**Scenario 1**: One infected patient introduced during the first day’s morning block; or
**Scenario 2**: One infected HCP introduced during the first day’s morning block.

As a baseline, we run simulations for 30 consecutive days for each of these 12 settings. We then run 30-day simulations evaluating each of the 6 NPIs individually. Finally, we evaluate the three intervention bundles Baseline+, Baseline++, and Baseline+++ described above.

Our Python code to generate contact networks and run our simulations along with extensive documentation is available on our github page [48], for free download and use.

## Results

### Contact networks

We gathered ten days of HCP movement and interaction data. Six days had 14.5–15 hours of observation (days 2, 6, 7, 8, 9, and 10), whereas the remaining four days had an average of 6.5 hours of observation. The results presented in the paper are based on Day 10 interaction data. Results based on other days of data are provided in Figs W-AP in the S1 Appendix. Fig 3 and Table 2 depict the static contact network *G* and provide summary statistics, respectively. Table 3 shows statistics of the temporal contact networks, averaged over all *d* and *t*. The network statistics of *G* and *G*^*d*,*t*^ reveal the high density of close range contacts among agents in the dialysis unit. For example, the average weighted degree of an HCP is 8.10 hrs per day (row 7 in Table 2), implying that on average an HCP in the dialysis unit has a total of 8.10 hrs of close contact with other dialysis unit agents in a day. Note that this quantity is high because agents typically have multiple simultaneous contacts. This is supported by the finding in Table 3 on temporal graphs *G*^*d*,*t*^ that shows that on average each HCP is in contact with more than 1.6 other agents per timestep.

**Table 2:** Network statistics of static graph *G*.

Number of nodes	51		
Number of edges	520		
Other statistics	mean	std. dev.	max
Degree	19.96	12.25	50
Weighted degree (hrs per day)	3.77	2.66	11.12
HCP degree	41.73	9.18	50
HCP weighted degree (hrs per day)	8.10	2.72	11.12
patient degree	13.98	1.44	17.00
patient weighted degree (hrs per day)	2.59	0.65	4.19
HCP-HCP edge weight (hrs per day)	0.41	0.26	1.11
HCP-patient edge weight (hrs per day)	0.13	0.08	0.43
patient-patient edge weight (hrs per day)	0.28	0.17	0.78

**Table 3:** Network statistics of temporal graph *G*^*d*,*t*^. The statistics shown here are obtained by averaging over all (approximately) 177K temporal graphs (177K ≈ 26 days × 6822 timesteps per day).

	mean	std dev	max
overall degree	1.760	0.640	2.744
HCP degree	1.635	0.396	2.117
patient degree	1.744	0.619	2.536

### Baseline simulations

We turn our attention now to the results of our simulations, focusing on worst case modeling assumptions (i.e., *R*_0_ = 3.0, and the *exp/exp (60%)* shedding model). We perform 500 replicates of each trial and report average values to account for stochastic effects in the simulation. Later, we summarize results obtained for both scenarios with different target *R*_0_ values and shedding models.

**Fig 4:**
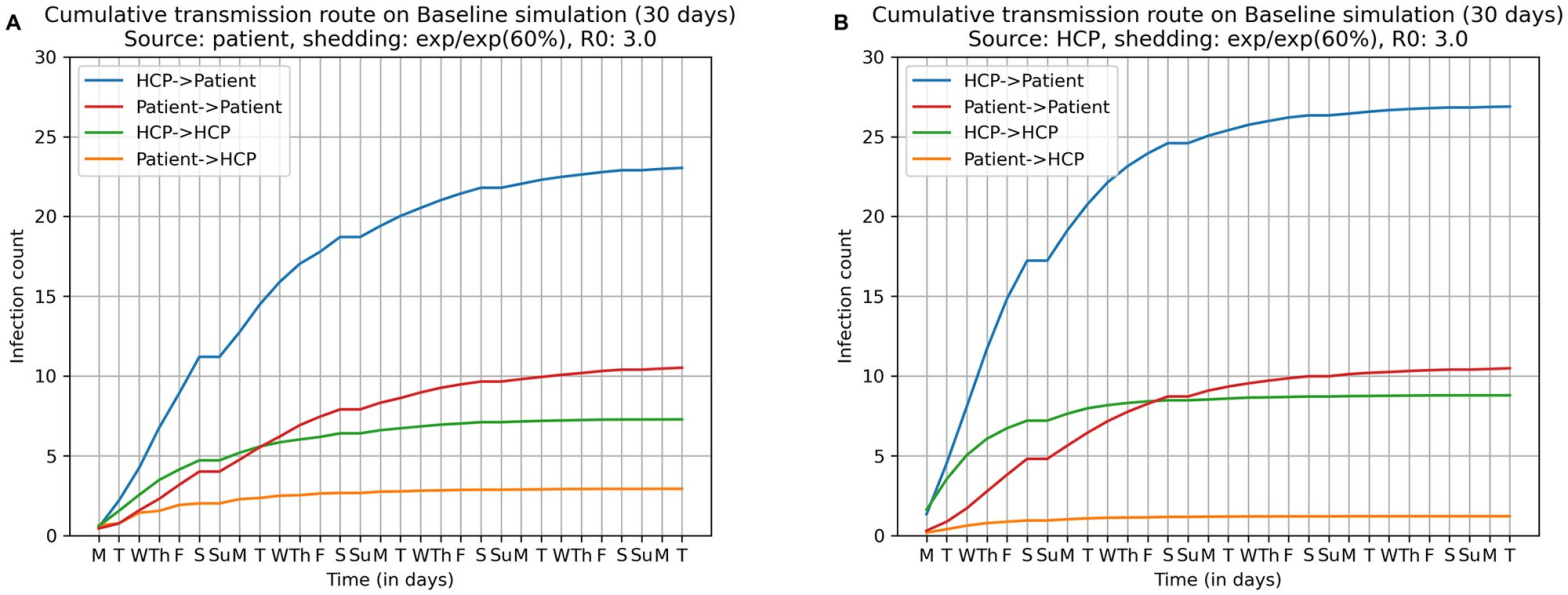
Cumulative distributions of transmission events over 30 days in Baseline simulation. **(A) Scenario 1: dialysis patient is the infection source and (B) Scenario 2: HCP is the infection source**. In both scenarios, the most frequent transmission route is from HCP → patient (52.7% in Scenario 1 and 56.8% in Scenario 2), followed by patient → patient (22.1% in Scenario 1 and 22.5% in Scenario 2), HCP → HCP (16.6% in Scenario 1 and 18.5% in Scenario 2), and a small fraction of patient → HCP transmission (6.7% in Scenario 1 and 2.6% in Scenario 2).

No interventions are imposed in the Baseline simulation: infected HCPs report for work as usual, and patients continue to dialyze in accordance with their original schedule even if infected. The high contact environment of the dialysis unit combined with the relatively high viral shedding rate of the shedding model ensures that almost everyone in the unit is infected within 30 days. The average (median) attack rates were 87.8% (94.1%) and 94.8% (96.1%) in Scenario 1 and Scenario 2 respectively. Furthermore, a substantial fraction of the simulations—45.8% and 53.6% in the two scenarios—resulted in a near 100% attack rate (attack rate of 96.1% or higher). The simulation also provides some insight into the underlying disease dynamics; this is shown in Fig 4. Independent of scenario and despite the fact that patients outnumber HCPs nearly 4:1, HCPs are the source of 69.3% of infections in Scenario 1 (infection source: dialysis patient; see Fig 4A) and 75.3% of the infections in Scenario 2 (infection source: HCP; see Fig 4B). We also note a surprisingly high number of patient → patient transmissions, a consequence of the physical proximity of dialysis stations. In both scenarios, there are very few patient → HCP transmissions. However, there are enough patient → HCP transmissions in Scenario 1, early in the simulation, e.g., on average 2 HCPs get infected by patients in the first week, that then allows HCPs to become main source of transmissions. This implies that in Scenario 1, patient → HCP transmissions might be the ones to most aggressively target via interventions. However, the situation in Scenario 2 is considerably more dire. In this scenario, almost immediately there are some HCP → HCP transmissions and HCP → patient transmissions. This means that by the time symptoms emerge in any infected agent, triggering the interventions in Baseline++ and Baseline+++, disease has already spread to a number of agents and is hard to subsequently contain.

### Effect of individual NPIs

**Fig 5:**
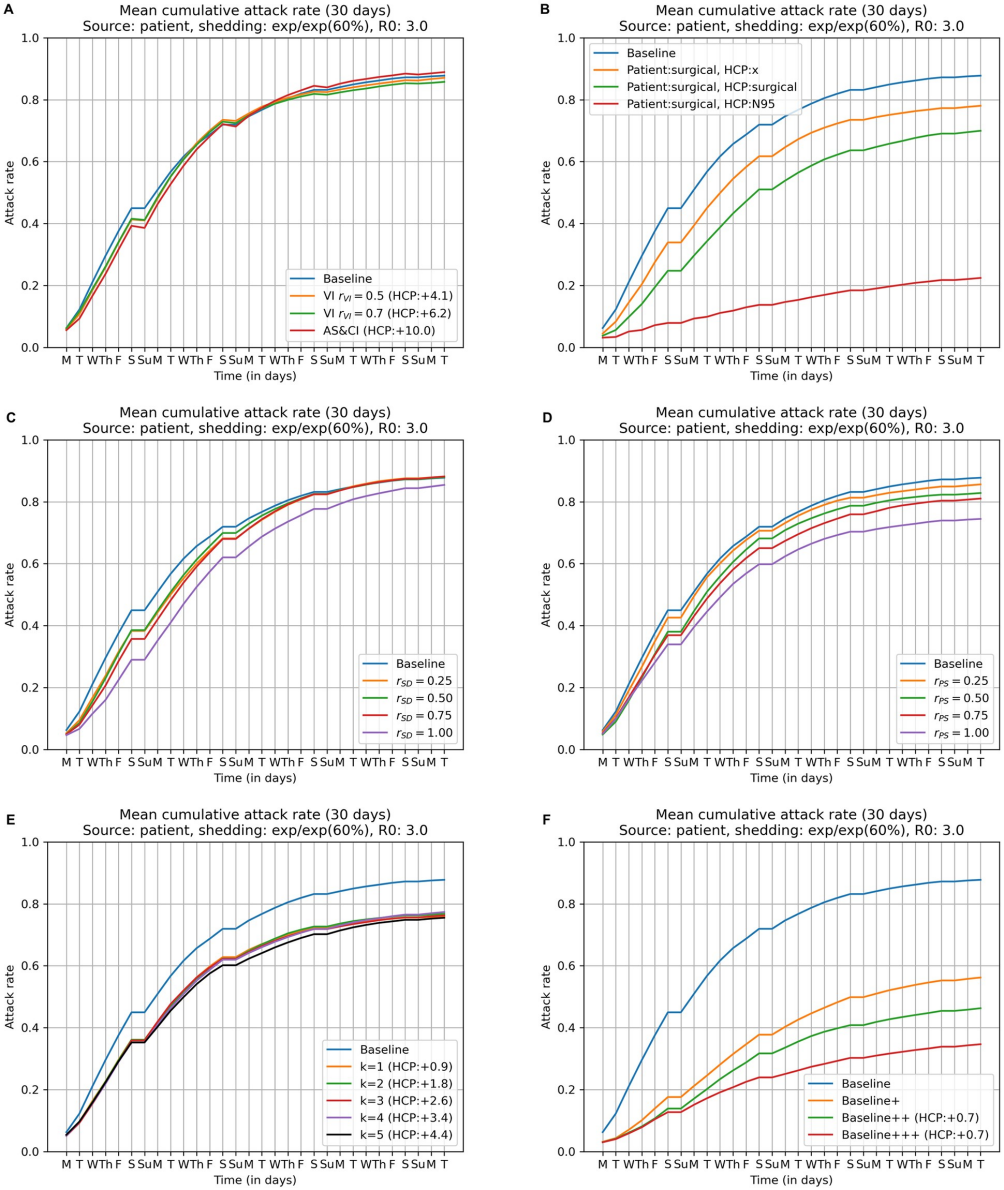
Attack rates for individual NPIs and selected NPIs in combination in Scenario 1 (infection source: Dialysis patient), *R*_0_ = 3, *exp/exp (60%)*. (A) Voluntary self-isolation vs. active syndromic surveillance and compulsory isolation. (B) Use of masks and respirators. (C) Improved social distancing among HCPs. (D) Increased physical separation of dialysis stations. (E) Patient isolation and preemptive isolation of exposed HCPs. (F) Combinations of inexpensive NPIs denoted Baseline+, Baseline++, and Baseline+++. Each figure displays cumulative attack rates as a function of simulation days. Each plot includes the Baseline curve for comparison; lower curves correspond to fewer infected agents. See text for descriptions of the individual NPIs.

**Fig 6:**
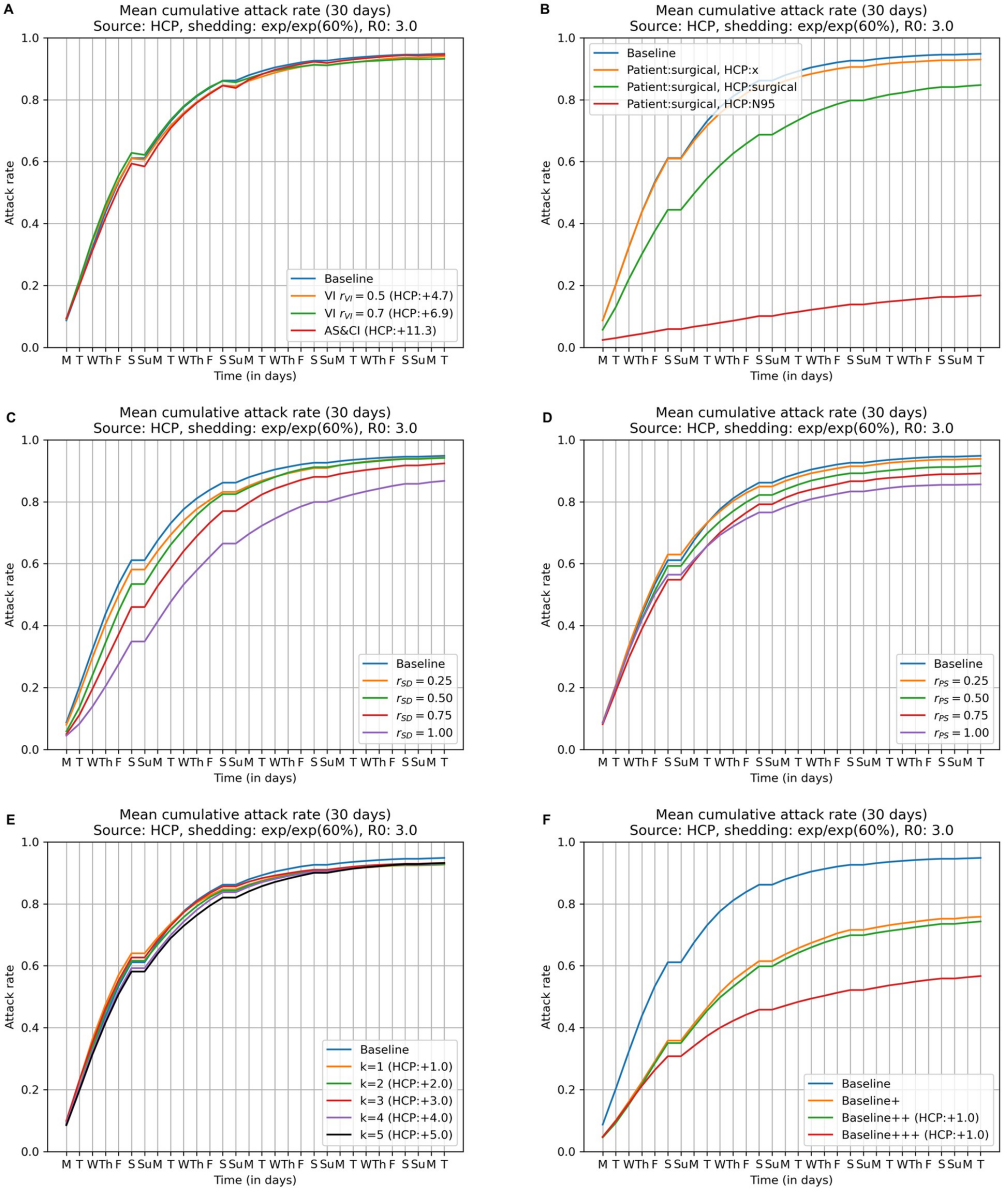
Attack rates for individual NPIs and selected NPIs in combination in Scenario 2 (infection source: HCP), *R*_0_ = 3, *exp/exp (60%)*. These are the same plots as in Fig 5, except that these are for Scenario 2.

Figs 5A and 6A compare the cumulative attack rates for voluntary self-isolation (*r*_*VI*_ ∈ {0.5, 0.7}) as well as active syndromic surveillance and compulsory isolation against that of the Baseline strategy (no interventions). These interventions have only minimal effect on the overall attack rate, but at a cost of employing a large number of additional substitute HCPs (replacement HCP counts are shown in parenthesis). For example, using the active syndromic surveillance and compulsory isolation intervention requires 10.0 additional substitute HCPs in Scenario 1 (see Fig 5A) and 11.3 in Scenario 2 (see Fig 6A). In Table A in S1 Appendix, we provide additional analyses on the voluntary self-isolation.

Figs 5B and 6B compares the cumulative attack rates when using masks and/or respirators with the Baseline strategy (no interventions) in Scenario 1 and 2 respectively. The three curves shown correspond to (*i*) patients wear surgical masks and HCPs are unmasked (78.0% and 93.0% overall attack rate in the two scenarios); (*ii*) patients wear surgical masks and HCPs wear surgical masks (70.0% and 84.7% overall attack rate in the two scenarios); and (*iii*) patients wear surgical masks and HCPs wear N95 respirators (22.4% and 16.8% overall attack rate in the two scenarios). Patients wearing surgical masks and HCPs wearing N95 respirators throughout their time in the dialysis unit has a significant effect on overall attack rate, leading to a 74.4% and 82.3% reduction in the two scenarios.

Figs 5C and 6C compare the cumulative attack rates observed with increasing social distancing among HCPs, again, in comparison with the Baseline strategy (no interventions). Recall the underlying model for this intervention is to remove a fraction *r*_*SD*_ ∈ {0.25, 0.5, 0.75, 1} of contacts that occur between pairs of HCPs when outside the immediate proximity of a dialysis station, while leaving HCP/patient and patient/patient contacts unchanged. These interventions do not have a significant effect on overall attack rates unless these pairs of HCP/HCP contacts get removed completely.

Increasing the physical separation between dialysis stations (Figs 5D and 6D) reduces opportunities for transmission not only between patients in adjacent chairs, but also with HCPs directly attending to either of the two stations. Recall that increasing the physical separation between dialysis stations is modeled by a parameter *r*_*PS*_ ∈ {0.25, 0.5, 0.75, 1} that denotes the fraction of relevant contacts that are removed. When compared with the Baseline strategy (no interventions), we see some reduction in overall attack rate in Scenario 1 (infection source: dialysis patient) as the degree of additional separation increases; complete separation reduces the Baseline strategy’s 87.8% overall attack rate to 74.5%, although physical space constraints may preclude achieving complete separation in any real application. We see a similar effect in Scenario 2, but the magnitude of the reduction is a little bit smaller compared to that of Scenario 1.

Figs 5E and 6E compare the effect of isolating the first patient with detectable symptoms and preemptively isolating the top *k* HCPs (*k* ∈ {1⋯5}) exposed to that patient with the Baseline strategy (no interventions). In Scenario 1 (infection source: dialysis patient), as the figure makes clear, the majority of the effect of this intervention occurs with *k* = 1, that is, preemptive isolation of the single most exposed HCP, which reduces the attack rate to 75.9%. In contrast, in Scenario 2 this intervention has no noticeable effect.

### Combining NPIs

Figs 5F and 6F compare three strategies that combine multiple NPIs with the Baseline strategy (no interventions). Because the combined interventions shown here (Baseline+, Baseline++, and Baseline+++) consist of increasingly “nested” sets of interventions, it is not entirely surprising that each strategy demonstrates increasing advantage, measured in terms of cumulative attack rates, with respect to the Baseline.

The Baseline+ strategy (surgical masks for HCPs and patients; improved social distancing among HCPs *r*_*SD*_ = 0.25; and increased physical separation between dialysis stations *r*_*PS*_ = 0.75) reduces the attack rate to 56.2% in Scenario 1 and to 75.8% in Scenario 2 from a Baseline attack rate of 87.8% and 94.8% in the two scenarios. The Baseline++ strategy, which adds patient isolation and preemptive isolation of a single exposed HCP to the Baseline+ strategy, reduces the attack rate to 46.5% in Scenario 1, but yields no improvement in the attack rate relative to Baseline+ in Scenario 2. Finally, Baseline+++, which adds 2 weeks of N95 usage for HCPs following the detection of the first symptomatic patient, produces an impressive 60.4% reduction in the Baseline strategy’s attack rate in Scenario 1 (to 34.8%), at a relatively modest cost of 0.7 replacement HCPs and a similarly modest number of N95 masks for HCPs only over a two week period. Moreover, unlike with the Baseline++ strategy, the Baseline+++ strategy also has a strong positive effect in Scenario 2, reducing the attack rate to 56.7% at a small cost of 1 replacement HCP.

**Fig 7:**
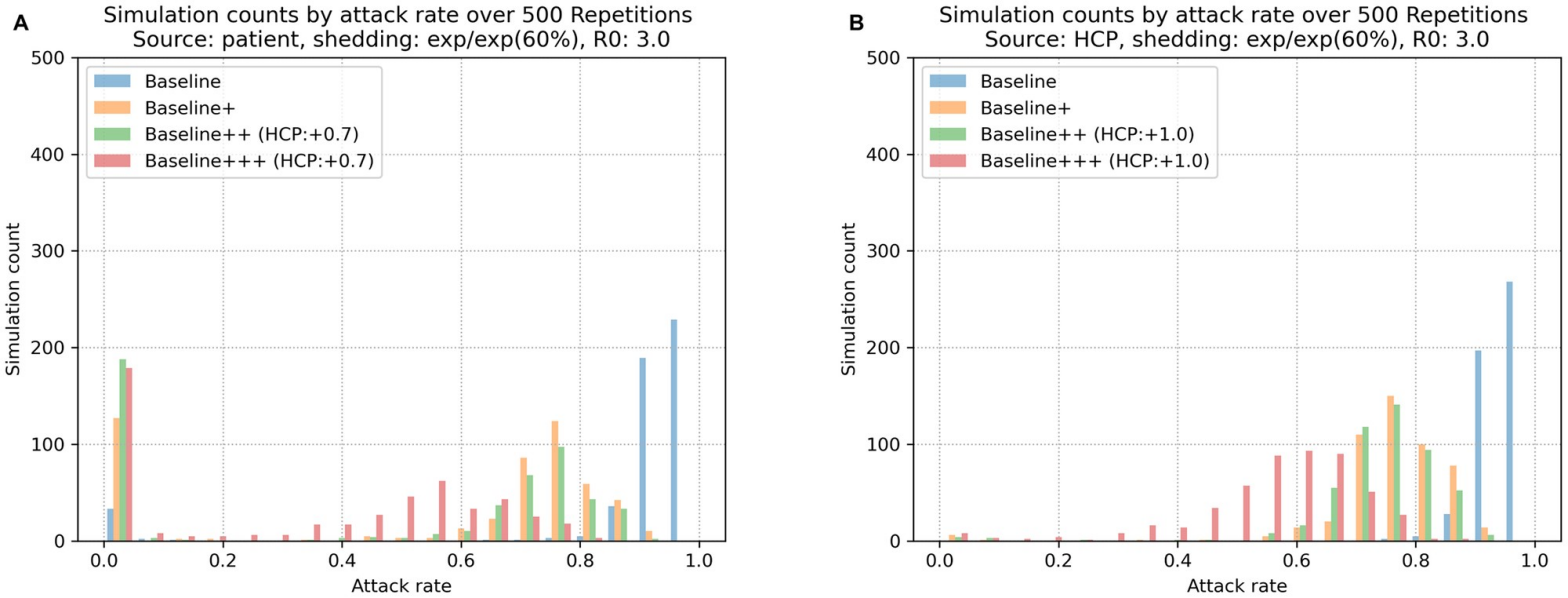
Frequency of replicates as a function of attack rates for different NPIs: (A) Scenario 1 and (B) Scenario 2. Each histogram displays the number of replicates (y axis; out of 500) with the specified overall attack rate. In Scenario 1, two peaks are commonly observed for each histogram; one where no transmission events occur (the infection is immediately quashed) and one indicating the fuller extent of an eventual outbreak. When no intervention is imposed (Baseline), only 31 simulations (6.2%) have no transmission events, while 83.6% (418) resulted in attack rates larger than 90%. In contrast, Baseline+++ and Baseline++ significantly increases the likelihood of observing no transmission events (162 and 175 simulations, 32.4% and 35.0%, respectively). In Scenario 2, even with interventions, infection is never quashed in any replicate. However, even in this scenario, there are no replicates with Baseline+++ interventions that have a near-100% attack rate. Furthermore, the frequency mass of the Baseline+++ intervention is substantially shifted to the left (towards lower attack rates) relative to the Baseline simulations.

Reporting the average attack rates obscures the distinction between replicates where the infection fails to spread beyond the first infected agent and those where the infection permeates the agent population. The histograms in Fig 7 makes the nature of this mixture explicit by showing the number of replicates (y axis; out of 500 total) that result in a given attack rate (x axis). What the plot for Scenario 1 (Fig 7A) makes clear is that more effective strategies (here, Baseline ≪ Baseline+ ≪ Baseline++ ≪ Baseline+++) affect both aspects of performance: it is both more likely that the infection is immediately quashed and that—when it does spread—the spread is decidedly more limited. So for the Baseline strategy, 83.6% of replicates (418) had attack rates larger than 90%, while only 6.2% of replicates (31) had no transmission events. Successively more effective strategies increased the number of quashed infections to a greater degree, with 32.4% of the Baseline+++ replicates (162) having no transmission events at all. Furthermore, the frequency mass of each strategy with interventions moved substantially to the left, relative to the immediately less effective strategy. In Scenario 2 (Fig 7B), the situation is not as positive mainly because despite the interventions, the infection is never quashed immediately. Some of the interventions that are effective in Scenario 1 (e.g., patient isolation and preemptive isolation) start too late for Scenario 2 and others (e.g., increased physical separation of dialysis units) have too little effect, given how many agents quickly become infected. Nevertheless, the frequency mass of the Baseline+ and Baseline++ strategies moved substantially to the left relative to the Baseline strategy and the frequency mass of the Baseline+++ strategy moved even more to the left relative to these less comprehensive strategies.

In Figs S-V in the S1 Appendix, we show all the above-mentioned results for the *exp/exp (20%)* shedding model, for both scenarios, and for *R*_0_ = 3.0. The symptom-based interventions (e.g. voluntary self-isolation, active syndromic surveillance, and isolating the first patient with detectable symptoms and preemptively isolating top *k* exposed HCPs) are more effective in *exp/exp (20%)* shedding model, because the total amount of shedding before symptom is less compared to the *exp/exp (60%)* shedding model. In Figs A-R in the S1 Appendix, we report results for the *exp/exp (60%)* shedding model for other values of *R*_0_, i.e., *R*_0_ ∈ {2.0, 2.5}. We also report results for the *exp/exp (60%)* shedding model with *R*_0_ = 3.0, using other values, 1.5 and 2.5, of the shedding ramp down parameter *γ*.

Table 4 summarizes attack rates for Baseline, Baseline+, Baseline++, and Baseline+++ for both scenarios, both shedding models, and three different target *R*_0_ values. As the combined interventions become increasingly sophisticated, the attack rates for Scenario 1 consistently drop regardless of shedding model and the target *R*_0_ value. The attack rates in Scenario 2, where the infection source is the HCP, were larger than their corresponding attack rates in Scenario 1. The attack rates of same set of interventions on the *exp/exp (60%)* shedding model is always larger than that of the *exp/exp (20%)* shedding model.

**Table 4:** Attack rates for combinations of NPIs under different modeling assumptions. “SM” is short for “shedding models”, “B” is short for “Baseline,” in Scenario 1 the infection source is a dialysis patient and in Scenario 2 the infection source is a HCP.

SM	*R*_0_	Scenario 1				Scenario 2			
		B	B+	B++	B+++	B	B+	B++	B+++
	2.0	62%	29%	19%	15%	82%	52%	51%	36%
20%	2.5	75%	45%	31%	22%	88%	65%	64%	46%
	3.0	84%	50%	41%	29%	93%	74%	72%	54%
	2.0	69%	32%	24%	17%	84%	54%	54%	39%
60%	2.5	83%	48%	37%	28%	91%	66%	65%	49%
	3.0	88%	56%	47%	35%	95%	76%	74%	57%

As a form of validation, we ran the same set of simulations using HCP movement and interaction patterns extracted from the other days data within our dataset. These are also reported in Figs W-AP in the S1 Appendix. And while the attack rates varied slightly due to minor differences in the number of HCPs and patients as well as their underlying contact patterns, we observed the same relationship between intervention strategy and reduced attack rates as we moved from the Baseline strategy towards Baseline+++.

## Discussion

Evidence suggests that viral respiratory infections like COVID-19 can spread very quickly in densely populated environments. For example, in early March 2020, 94 workers in a South Korean call center (43.5% of the total) were infected, notwithstanding that immediate action was taken to close the call center when the outbreak was first reported [49]. Dialysis units are also good examples of densely populated environments, where frequent close contacts occur primarily between patients and HCPs on a recurring basis. Morover, not only are dialysis settings particularly suited to the transmission of COVID-19, but dialysis patients are particularly susceptible to adverse effects of the infection thanks to their underlying health concerns. A recent study examining clinical course and outcomes for 36 COVID-19 positive hemodialysis patients in Spain found that 18 of the patients eventually suffered degraded clinical status and 11 patients died outright, a 30.5% mortality rate [12]. Our simulations support this evidence and show how a single infection in a dialysis unit can quickly lead to nearly universal infection across the unit due largely to the intensity of contacts among HCPs and patients.

In summary, in both scenarios, the most effective individual NPI is the continuous use of surgical masks by patients and N95 respirators by HCPs. In Scenario 1, two other interventions, namely (i) increased physical separation between dialysis stations and (ii) patient isolation and preemptive isolation of a few exposed HCPs are moderately successful, whereas in Scenario 2, (i) increased physical separation between dialysis stations and (ii) social distancing among HCPs are somewhat effective.

The relatively small impact on attack rates for voluntary self-isolation and active syndromic surveillance and compulsory isolation is likely attributable to the high rate of viral shedding prior to emergence of symptoms in the *exp/exp (60%)* shedding model. As we experience shortages of available HCPs in the midst of the COVID-19 outbreak [50], substituting a large number of HCPs may not be a viable option, especially given the relatively low reduction in attack rate. As a result, we conclude that voluntary self-isolation and active syndromic surveillance and compulsory isolation are not particularly effective NPIs in the dialysis unit.

The usage of masks, especially for the case where patients wear surgical masks and HCPs wear N95 respirators, is substantially better than the rest of the NPIs. If the continuous use of N95 respirators is feasible, then the consistent use of surgical masks for patients and N95 respirators for HCPs is clearly an intervention that should be implemented. Unfortunately, due to the sharp increase in demand for N95 respirators [51], it in unclear if this is a feasible intervention at a typical dialysis unit. This observation inspired our Baseline+++ strategy, which includes a more limited deployment of N95 respirators once the first symptomatic patient is detected (see Section “Combining NPIs”).

The strategy of isolating the first patient with detectable symptoms followed by isolating preemtive isolation of exposed HCPs performs well in Scenario 1. Replacing more HCPs had relatively little effect on the attack rate, while concomitantly raising the cost in terms of additional HCPs, which, as noted previously, may not be feasible. Moreover, with this intervention it is at least technically possible that one might isolate a healthy HCP while importing an infected substitute. Notice that this intervention had no noticeable effect in Scenario 2. This can be explained by the transmission dynamics shown in Fig 4. By the time the first patient shows any symptoms, the disease has spread from the infection source (an HCP) to several other agents. At this point, isolating the first patient with detectable symptoms and preemptively isolating a few HCPs is “too little, too late.”

The primary message of this paper is that a strategy combining simple, inexpensive, and practical interventions can substantially reduce the overall attack rate. The Baseline+ strategy (surgical masks for HCPs and patients; improved social distancing among HCPs *r*_*SD*_ = 0.25; and increased physical separation between dialysis stations *r*_*PS*_ = 0.75) shows substantial reduction in the attack rate in both scenarios. However, the fact that the attack rate is not reduced as much in Scenario 2 could be due to the higher baseline attack rate observed in Scenario 2. In the Baseline++ strategy (Baseline+; patient isolation and preemptive isolation of a single exposed HCP), the difference between the impressive reduction in attack rate observed in Scenario 1 and the negligible effect observed in Scenario 2 is likely due to the fact that patient isolation and preemptive isolation of a single exposed HCP is “too little, too late” given how much the disease has spread before the first patient shows symptoms.

The attack rates in Scenario 2 were larger than their corresponding attack rates in Scenario 1, perhaps indicating the initial patient-to-HCP jump in Scenario 1 presents a bottleneck to broader transmission. Supporting this particular intuition is the fact that, in Scenario 2, attack rates for Baseline++ are similar to those of Baseline+, indicating that isolating the first symptomatic patient and preemptive isolation the corresponding most exposed HCP does not have much effect. The attack rates in *exp/exp (60%)* shedding model were larger than their corresponding attack rates of the same set of interventions in *exp/exp (20%)* shedding model. Although the volume of shedding is set to be the same for the two shedding models, the difference in the dynamics comes from the percentage of presymptomatic shedding.

The Baseline+++ intervention (Baseline++; use of N95 respirators by all HCPs for 2 weeks following detection of the first symptomatic patient) performed well in the simulations reported here. Note that the primary interventions in Baseline+++ are all relatively inexpensive by design: relatively more expensive interventions (such as preemptive HCP isolation, or the use of N95 respirators) are applied in a targeted fashion only when an infection is first detected. Such targeting helps to reduce overall cost and make the Baseline+++ strategy economically feasible. Implementing Baseline+++ in dialysis unit modeled here would, in practice, require one additional temporary HCP for 12 days (Sundays excluded) and 132 N95 respirators are needed (11 HCPs, 12 days).

A second interesting finding, clearly evident in Fig 7, is that an infection in the dialysis unit can evolve in two distinct ways: it is either immediately suppressed, or it eventually overtakes nearly the entire unit. Once the infection spreads to other agents, even the combination of interventions in the Baseline+++ strategy cannot effectively dampen the outbreak. This observation highlights the critical temporal aspects involved in mounting an adequate response, and suggests that rigorous but targeted practices applied to prevent subsequent transmission beyond the first patient may be the key to preventing a catastrophic outbreak of COVID19 in closed, high-contact systems like our dialysis unit. Here, we focused on cost-efficient interventions that can be easily implemented in practice. However, if COVID-19 testing were a viable option in terms of cost, an even more rigorous program of targeted interventions could be deployed with confirmed COVID-19 patients. Indeed, a recent study of a COVID-19 outbreak in a dialysis center (Wuhan, China; January—March 2020) provides some evidence to support this claim [52]. The study reports that 18.26% of dialysis patients and 12.12% of HCPs in their facility were diagnosed with COVID-19. This attack rate is much lower than in our simulations, but we note that they followed a rigorous program of interventions guided by an aggressive in-house COVID-19 testing program. Within one week of the first confirmed COVID-19 case, all HCPs wore full protective gear, including waterproof disposable gowns, caps, gloves, face shields, and N95 respirators. In addition, an extensive cleaning and disinfection program was implemented. Finally, during the screening period, all infected patients and HCPs were isolated or transferred to other hospitals.

In our modeling and simulations, we used *R*_0_ values in the range [2, 3]. These were based on estimates of *R*_0_ for COVID-19 in the general population. There are almost no prior studies that can be used to estimate an *R*_0_ value or an attack rate in a dialysis unit. One exception is a study that performed contact tracing of a COVID-19 outbreak at a single hemodialysis center in Toronto, Canada [53]. There were two seed cases, a patient and a HCP. The seed patient infected one other patient and the seed HCP infected six others (4 patients and 2 HCPs). This can be interpreted as yielding an average *R*_0_ of 3.5 (2 seeds leading to 7 secondary cases). This trend—of an infected patient leading to fewer secondary cases compared to an infected HCP—is what we observe in our simulations as well. The overall attack rate reported in this study was low (0.07), due to an extensive intervention upon detection of a few COVID-19 cases. These interventions included universal droplet and contact precautions including gloves, face shields, surgical masks, and isolation gowns, that were imposed on top of the interventions that were already in place (e.g., physical distancing, universal masking for staff, and screening for symptoms for patients).

In addition to COVID-19, dialysis patients are at risk for other respiratory infections spread by droplets. Accordingly, our results have implications for how to prevent the spread of other respiratory infections. For example, the acquisition of MERS, a virus similar to COVID-19, was also associated with treatment in dialysis centers [54, 55]. In addition, every year, hemodialysis patients are at risk for acquiring influenza, and they are more likely to suffer from worse influenza-associated outcomes than patients not on hemodialysis. Also, they are less likely to benefit from the influenza vaccine than people not undergoing hemodialysis [16].

Some of our results also have implications for healthcare-associated infections spread by close contact (e.g., multi-drug-resistant infections spread by the hands of healthcare workers). Specifically, patients on hemodialysis are at risk for a wide range of multidrug resistant organisms (MDROs) including MRSA, vancomycin-resistant Enterococcus (VRE), and carbapenem-resistant Enterobacteriaceae (CRE) [3] and MDRO colonization rates are extremely high [56–59].

A comprehensive study on the comparison of dialysis facilities showed that the mean of the number of stations over 5,193 facilities was 18.9 (std dev 7.8) [60]. So the UIHC dialysis center is somewhat smaller than average. Although there are larger and smaller dialysis units, the demands of care require a specific and fairly constant distribution of interactions with members of the healthcare staff on average over a typical shift. For instance, there was a small variance in the dialysis sessions of 3.8 (0.3) hours across all facilities as well as the number of dialysis sessions per week per patient 3.0 (0.2). (see Table 4.1 from [60]). As far as we know, dialysis centers have a similar physical layout: nurses’ station in the central area and the stations placed around the outer perimeter of the unit. Even if dialysis units have radically different layout, HCP-patient contact patterns and patient-patient contact patterns will likely remain unchanged. HCP-HCP contact patterns may change, but as seen in Fig 4 HCP-patient contacts and patient-patient contacts play a dominant role in transmissions. The fact that COVID-19 is primarily spread by airborne respiratory droplets also means that the physical arrangement of surfaces in the dialysis unit does not play as much of a role as it might in the spread of an infection such as MRSA whose transmission is fomite-based [20, 61, 62]. Thus, we anticipate that our results and findings based on our simulations should be generalizable to other dialysis settings.

Our results also have broad applications for other healthcare settings beyond dialysis centers where patients repeatedly receive care for prolonged periods. Specific examples include chemotherapy units where patients come regularly and sit in chairs for several hours at a time. In addition, patients receiving parenteral antibiotics through peripherally inserted central catheter (PICC) lines arrive daily and can stay up to several hours, receiving monitoring and care from healthcare professionals. Like dialysis, for both chemotherapy and parenteral antibiotic treatment, the longest durations of interactions between healthcare professionals and the patients under their care occur at the beginning and end of a therapy session.

Our work is subject to a few limitations. First, although we have explored interventions that reduce HCP-HCP contacts and patient-patient contacts, we did not explore interventions that reduce or modify HCP-patient contacts. Since most of these result from direct patient care activity, it would not be reasonable to expect a uniform reduction in these contacts as we did for the other types of contacts. We could, however, consider reorganizing dialysis schedules so as to reduce the diversity of HCP-patient contacts by, *e.g*., assigning each patient to a unique HCP in order to reduce redundant transmission paths. Second, we have limited our model to consider only person-to-person respiratory droplet transmission of the virus. However, aerosol and fomite transmission are also plausible, as SARS-CoV-2 can survive in aerosols for several hours and on surfaces for several days [63]. Detailed modeling of these transmission routes will require more data, such as viral survival rates in the air or on different types of surfaces. But these other transmission routes will also almost certainly suggest other interventions such as improvements in hand-hygiene protocols or additional cleaning practices which may in turn become important elements of new cost-effective practical interventions. Third, we have limited our focus to transmissions within the dialysis unit. A recent study pointed out that sharing health-care transportation to the dialysis unit is a significant risk factor for COVID-19 infection [64]. Our paper does not consider the role of such interactions.

## Supporting information

S1 Appendix A document that contains results on additional experiments. In the paper, we showed simulation results focusing on the worst case modeling assumptions (i.e., *R*_0_ = 3.0, and the *exp/exp (60%)* shedding model). Here, in the Appendix, we show results on other settings of the parameters as follows:
*exp/exp (60%)* shedding model, *R*_0_ ∈ {2.0, 2.5}*exp/exp (60%)* shedding model, *R*_0_ = 3.0, *γ* ∈ {1.5, 2.5}*exp/exp (20%)* shedding model, *R*_0_ = 3.0Additional analyses on voluntary self-isolation*exp/exp (60%)* shedding model, *R*_0_ = 3.0, on the other days of data
**Fig A. Cumulative distributions of transmission events over 30 days in Baseline simulation on *R*_0_ = 2.0, and the *exp/exp (60%)***. (A) Scenario 1: dialysis patient is the infection source. (B) Scenario 2: HCP is the infection source. **Fig B. Frequency of replicates as a function of attack rates for different NPIs on *R*_0_ = 2.0, and the *exp/exp (60%)***. (A) Scenario 1. (B) Scenario 2. **Fig C. Attack rates for individual NPIs and selected NPIs in combination in Scenario 1 (infection source: dialysis patient), *R*_0_ = 2.0, *exp/exp (60%)***. (A) Voluntary self-isolation vs. active syndromic surveillance and compulsory isolation. (B) Use of masks and respirators. (C) Improved social distancing among HCPs. (D) Increased physical separation of dialysis stations. (E) Patient isolation and preemptive isolation of exposed HCPs. (F) Combinations of inexpensive NPIs. **Fig D. Attack rates for individual NPIs and selected NPIs in combination in Scenario 1 (infection source: HCP), *R*_0_ = 2.0, *exp/exp (60%)***. (A) Voluntary self-isolation vs. active syndromic surveillance and compulsory isolation. (B) Use of masks and respirators. (C) Improved social distancing among HCPs. (D) Increased physical separation of dialysis stations. (E) Patient isolation and preemptive isolation of exposed HCPs. (F) Combinations of inexpensive NPIs. **Fig E. Cumulative distributions of transmission events over 30 days in Baseline simulation on *R*_0_ = 2.5, and the *exp/exp (60%)***. (A) Scenario 1: dialysis patient is the infection source. (B) Scenario 2: HCP is the infection source. **Fig F. Frequency of replicates as a function of attack rates for different NPIs on *R*_0_ = 2.5, and the *exp/exp (60%)***. (A) Scenario 1. (B) Scenario 2. **Fig G. Attack rates for individual NPIs and selected NPIs in combination in Scenario 1 (infection source: dialysis patient), *R*_0_ = 2.5, *exp/exp (60%)***. (A) Voluntary self-isolation vs. active syndromic surveillance and compulsory isolation. (B) Use of masks and respirators. (C) Improved social distancing among HCPs. (D) Increased physical separation of dialysis stations. (E) Patient isolation and preemptive isolation of exposed HCPs. (F) Combinations of inexpensive NPIs. **Fig H. Attack rates for individual NPIs and selected NPIs in combination in Scenario 1 (infection source: HCP), *R*_0_ = 2.5, *exp/exp (60%)***. (A) Voluntary self-isolation vs. active syndromic surveillance and compulsory isolation. (B) Use of masks and respirators. (C) Improved social distancing among HCPs. (D) Increased physical separation of dialysis stations. (E) Patient isolation and preemptive isolation of exposed HCPs. (F) Combinations of inexpensive NPIs. **Fig I. Viral shedding models**. The bar charts show the shedding level of an infected individual whose presymptomatic period and symptomatic period are 6 and 7 days, respectively. The percentage in the parenthesis of each model corresponds to the percentage of shedding during the presymptomatic state (*P*) relative to the total shedding volume. The peak shedding level differs in the two models, but the total shedding volume remains the same. The shedding models are calibrated by setting the shedding ramp down parameter of the *exp/exp (20%)* shedding model *γ* = 1.5. **Fig J. Cumulative distributions of transmission events over 30 days in Baseline simulation on *R*_0_ = 3.0, and the *exp/exp (60%)* shedding model where *γ* = 1.5**. (A) Scenario 1: dialysis patient is the infection source. (B) Scenario 2: HCP is the infection source. **Fig K. Frequency of replicates as a function of attack rates for different NPIs on *R*_0_ = 3.0, and the *exp/exp (60%)* shedding model where *γ* = 1.5**. (A) Scenario 1. (B) Scenario 2. **Fig L. Attack rates for individual NPIs and selected NPIs in combination in Scenario 1 (infection source: dialysis patient), *R*_0_ = 3.0, *exp/exp (60%)*, where *γ* = 1.5**. (A) Voluntary self-isolation vs. active syndromic surveillance and compulsory isolation. (B) Use of masks and respirators. (C) Improved social distancing among HCPs. (D) Increased physical separation of dialysis stations. (E) Patient isolation and preemptive isolation of exposed HCPs. (F) Combinations of inexpensive NPIs. **Fig M. Attack rates for individual NPIs and selected NPIs in combination in Scenario 1 (infection source: HCP), *R*_0_ = 3.0, *exp/exp (60%)*, where *γ* = 1.5**. (A) Voluntary self-isolation vs. active syndromic surveillance and compulsory isolation. (B) Use of masks and respirators. (C) Improved social distancing among HCPs. (D) Increased physical separation of dialysis stations. (E) Patient isolation and preemptive isolation of exposed HCPs. (F) Combinations of inexpensive NPIs. **Fig N. Viral shedding models**. The bar charts show the shedding level of an infected individual whose presymptomatic period and symptomatic period are 6 and 7 days, respectively. The percentage in the parenthesis of each model corresponds to the percentage of shedding during the presymptomatic state (*P*) relative to the total shedding volume. The peak shedding level differs in the two models, but the total shedding volume remains the same. The shedding models are calibrated by setting the shedding ramp down parameter of the *exp/exp (20%)* shedding model *γ* = 2.5. **Fig O. Cumulative distributions of transmission events over 30 days in Baseline simulation on *R*_0_ = 3.0, and the *exp/exp (60%)* shedding model where *γ* = 2.5**. (A) Scenario 1: dialysis patient is the infection source. (B) Scenario 2: HCP is the infection source. **Fig P. Frequency of replicates as a function of attack rates for different NPIs on *R*_0_ = 3.0, and the *exp/exp (60%)* shedding model where *γ* = 2.5**. (A) Scenario 1. (B) Scenario 2. **Fig Q. Attack rates for individual NPIs and selected NPIs in combination in Scenario 1 (infection source: dialysis patient), *R*_0_ = 3.0, *exp/exp (60%)*, where *γ* = 2.5**. (A) Voluntary self-isolation vs. active syndromic surveillance and compulsory isolation. (B) Use of masks and respirators. (C) Improved social distancing among HCPs. (D) Increased physical separation of dialysis stations. (E) Patient isolation and preemptive isolation of exposed HCPs. (F) Combinations of inexpensive NPIs. **Fig R. Attack rates for individual NPIs and selected NPIs in combination in Scenario 1 (infection source: HCP), *R*_0_ = 3.0, *exp/exp (60%)*, where *γ* = 2.5**. (A) Voluntary self-isolation vs. active syndromic surveillance and compulsory isolation. (B) Use of masks and respirators. (C) Improved social distancing among HCPs. (D) Increased physical separation of dialysis stations. (E) Patient isolation and preemptive isolation of exposed HCPs. (F) Combinations of inexpensive NPIs. **Fig S. Cumulative distributions of transmission events over 30 days in Baseline simulation on *R*_0_ = 3.0, and the *exp/exp (20%)* shedding model**. (A) Scenario 1: dialysis patient is the infection source. (B) Scenario 2: HCP is the infection source. **Fig T. Frequency of replicates as a function of attack rates for different NPIs on *R*_0_ = 3.0, and the *exp/exp (20%)* shedding model**. (A) Scenario 1. (B) Scenario 2. **Fig U. Attack rates for individual NPIs and selected NPIs in combination in Scenario 1 (infection source: dialysis patient), *R*_0_ = 3.0, *exp/exp (20%)***. (A) Voluntary self-isolation vs. active syndromic surveillance and compulsory isolation. (B) Use of masks and respirators. (C) Improved social distancing among HCPs. (D) Increased physical separation of dialysis stations. (E) Patient isolation and preemptive isolation of exposed HCPs. (F) Combinations of inexpensive NPIs. **Fig V. Attack rates for individual NPIs and selected NPIs in combination in Scenario 1 (infection source: HCP), *R*_0_ = 3.0, *exp/exp (20%)***. (A) Voluntary self-isolation vs. active syndromic surveillance and compulsory isolation. (B) Use of masks and respirators. (C) Improved social distancing among HCPs. (D) Increased physical separation of dialysis stations. (E) Patient isolation and preemptive isolation of exposed HCPs. (F) Combinations of inexpensive NPIs. **Fig W. Contact network *G* on Day 2**. We gathered ten days of HCP movement and interaction data where six days had 14.5-15 hours of observation (Day 2, Day 6, Day 7, Day 8, Day 9, and Day 10). HCP nodes, MWF patient nodes, and TThS patient nodes are depicted in blue, chocolate, and burlywood colors, respectively. We observe a total population of 42 agents (14 HCPs and 28 patients). Contacts within the same type of nodes are represented as edges colored according to node type. Grey colored edges represent HCP-patient contacts. The thickness of the edge corresponds to the contact duration. **Fig X. Cumulative distributions of transmission events over 30 days in Baseline simulation on Day 2 and *R*_0_ = 3.0**. (A) the *exp/exp (20%)* shedding model on Scenario 1. (B) the *exp/exp (20%)* shedding model on Scenario 2. (C) the *exp/exp (60%)* shedding model on Scenario 1. (D) the *exp/exp (60%)* shedding model on Scenario 2. **Fig Y. Attack rates for combinations of inexpensive NPIs on Day 2, *R*_0_ = 3.0**. (A) the *exp/exp (20%)* shedding model on Scenario 1. (B) the *exp/exp (20%)* shedding model on Scenario 2. (C) the *exp/exp (60%)* shedding model on Scenario 1. (D) the *exp/exp (60%)* shedding model on Scenario 2. **Fig Z. Frequency of replicates as a function of attack rates for combinations of inexpensive NPIs on Day 2, *R*_0_ = 3.0**. (A) the *exp/exp (20%)* shedding model on Scenario 1. (B) the *exp/exp (20%)* shedding model on Scenario 2. (C) the *exp/exp (60%)* shedding model on Scenario 1. (D) the *exp/exp (60%)* shedding model on Scenario 2. **Fig AA. Contact network *G* on Day 6**. We gathered ten days of HCP movement and interaction data where six days had 14.5-15 hours of observation (Day 2, Day 6, Day 7, Day 8, Day 9, and Day 10). HCP nodes, MWF patient nodes, and TThS patient nodes are depicted in blue, chocolate, and burlywood colors, respectively. We observe a total population of 47 agents (13 HCPs and 34 patients). **Fig AB. Cumulative distributions of transmission events over 30 days in Baseline simulation on Day 6 and *R*_0_ = 3.0**. (A) the *exp/exp (20%)* shedding model on Scenario 1. (B) the *exp/exp (20%)* shedding model on Scenario 2. (C) the *exp/exp (60%)* shedding model on Scenario 1. (D) the *exp/exp (60%)* shedding model on Scenario 2. **Fig AC. Attack rates for combinations of inexpensive NPIs on Day 6, *R*_0_ = 3.0**. (A) the *exp/exp (20%)* shedding model on Scenario 1. (B) the *exp/exp (20%)* shedding model on Scenario 2. (C) the *exp/exp (60%)* shedding model on Scenario 1. (D) the *exp/exp (60%)* shedding model on Scenario 2. **Fig AD. Frequency of replicates as a function of attack rates for combinations of inexpensive NPIs on Day 6, *R*_0_ = 3.0**. (A) the *exp/exp (20%)* shedding model on Scenario 1. (B) the *exp/exp (20%)* shedding model on Scenario 2. (C) the *exp/exp (60%)* shedding model on Scenario 1. (D) the *exp/exp (60%)* shedding model on Scenario 2. **Fig AE. Contact network *G* on Day 7**. We gathered ten days of HCP movement and interaction data where six days had 14.5-15 hours of observation (Day 2, Day 6, Day 7, Day 8, Day 9, and Day 10). HCP nodes, MWF patient nodes, and TThS patient nodes are depicted in blue, chocolate, and burlywood colors, respectively. We observe a total population of 46 agents (10 HCPs and 36 patients). **Fig AF. Cumulative distributions of transmission events over 30 days in Baseline simulation on Day 7 and *R*_0_ = 3.0**. (A) the *exp/exp (20%)* shedding model on Scenario 1. (B) the *exp/exp (20%)* shedding model on Scenario 2. (C) the *exp/exp (60%)* shedding model on Scenario 1. (D) the *exp/exp (60%)* shedding model on Scenario 2. **Fig AG. Attack rates for combinations of inexpensive NPIs on Day 7, *R*_0_ = 3.0**. (A) the *exp/exp (20%)* shedding model on Scenario 1. (B) the *exp/exp (20%)* shedding model on Scenario 2. (C) the *exp/exp (60%)* shedding model on Scenario 1. (D) the *exp/exp (60%)* shedding model on Scenario 2. **Fig AH. Frequency of replicates as a function of attack rates for combinations of inexpensive NPIs on Day 7, *R*_0_ = 3.0**. (A) the *exp/exp (20%)* shedding model on Scenario 1. (B) the *exp/exp (20%)* shedding model on Scenario 2. (C) the *exp/exp (60%)* shedding model on Scenario 1. (D) the *exp/exp (60%)* shedding model on Scenario 2. **Fig AI. Contact network *G* on Day 8**. We gathered ten days of HCP movement and interaction data where six days had 14.5-15 hours of observation (Day 2, Day 6, Day 7, Day 8, Day 9, and Day 10). HCP nodes, MWF patient nodes, and TThS patient nodes are depicted in blue, chocolate, and burlywood colors, respectively. We observe a total population of 45 agents (11 HCPs and 34 patients). **Fig AJ. Cumulative distributions of transmission events over 30 days in Baseline simulation on Day 8 and *R*_0_ = 3.0**. (A) the *exp/exp (20%)* shedding model on Scenario 1. (B) the *exp/exp (20%)* shedding model on Scenario 2. (C) the *exp/exp (60%)* shedding model on Scenario 1. (D) the *exp/exp (60%)* shedding model on Scenario 2. **Fig AK. Attack rates for combinations of inexpensive NPIs on Day 8, *R*_0_ = 3.0**. (A) the *exp/exp (20%)* shedding model on Scenario 1. (B) the *exp/exp (20%)* shedding model on Scenario 2. (C) the *exp/exp (60%)* shedding model on Scenario 1. (D) the *exp/exp (60%)* shedding model on Scenario 2. **Fig AL. Frequency of replicates as a function of attack rates for combinations of inexpensive NPIs on Day 8, *R*_0_ = 3.0**. (A) the *exp/exp (20%)* shedding model on Scenario 1. (B) the *exp/exp (20%)* shedding model on Scenario 2. (C) the *exp/exp (60%)* shedding model on Scenario 1. (D) the *exp/exp (60%)* shedding model on Scenario 2. **Fig AM. Contact network *G* on Day 9**. We gathered ten days of HCP movement and interaction data where six days had 14.5-15 hours of observation (Day 2, Day 6, Day 7, Day 8, Day 9, and Day 10). HCP nodes, MWF patient nodes, and TThS patient nodes are depicted in blue, chocolate, and burlywood colors, respectively. We observe a total population of 39 agents (13 HCPs and 26 patients). **Fig AN. Cumulative distributions of transmission events over 30 days in Baseline simulation on Day 9 and *R*_0_ = 3.0**. (A) the *exp/exp (20%)* shedding model on Scenario 1. (B) the *exp/exp (20%)* shedding model on Scenario 2. (C) the *exp/exp (60%)* shedding model on Scenario 1. (D) the *exp/exp (60%)* shedding model on Scenario 2. **Fig AO. Attack rates for combinations of inexpensive NPIs on Day 9, *R*_0_ = 3.0**. (A) the *exp/exp (20%)* shedding model on Scenario 1. (B) the *exp/exp (20%)* shedding model on Scenario 2. (C) the *exp/exp (60%)* shedding model on Scenario 1. (D) the *exp/exp (60%)* shedding model on Scenario 2. **Fig AP. Frequency of replicates as a function of attack rates for combinations of inexpensive NPIs on Day 9, *R*_0_ = 3.0**. (A) the *exp/exp (20%)* shedding model on Scenario 1. (B) the *exp/exp (20%)* shedding model on Scenario 2. (C) the *exp/exp (60%)* shedding model on Scenario 1. (D) the *exp/exp (60%)* shedding model on Scenario 2. **Table A. Attack rates for voluntary isolation compliance rate *r*_*VI*_∈ {0.4, 0.5, 0.6, 0.7, 0.8} under different modeling assumptions**. “SM” is short for “shedding models”, “B” is short for “Baseline,” in Scenario 1 the infection source is a dialysis patient and in Scenario 2 the infection source is a HCP.
(PDF)
