# Supplementary material for: COVID-19 modeling and non-pharmaceutical interventions in an outpatient dialysis unit
Source: PLoS Comput Biol. 2021 Jul 8;17(7):e1009177. doi: 10.1371/journal.pcbi.1009177 (PMC8291695; doi:10.1371/journal.pcbi.1009177)
Supplement: S1 Appendix — In the paper, we showed simulation results focusing on the worst case modeling assumptions (i.e., R0 = 3.0, and the exp/exp (60%) shedding model). Here, in the Appendix, we show results on other settings of the parameters as follows: exp/exp (60%) shedding model, R0 ∈ {2.0, 2.5}exp/exp (60%) shedding model, R0 = 3.0, γ ∈ {1.5, 2.5}exp/exp (20%) shedding model, R0 = 3.0Additional analyses on voluntary self-isolationexp/exp (60%) shedding model, R0 = 3.0, on the other days of data Fig A. Cumulative distributions of transmission events over 30 days in Baseline simulation on R0 = 2.0, and the exp/exp (60%). (A) Scenario 1: dialysis patient is the infection source. (B) Scenario 2: HCP is the infection source. Fig B. Frequency of replicates as a function of attack rates for different NPIs on R0 = 2.0, and the exp/exp (60%). (A) Scenario 1. (B) Scenario 2. Fig C. Attack rates for individual NPIs and selected NPIs in combination in Scenario 1 (infection source: dialysis patient), R0 = 2.0, exp/exp (60%). (A) Voluntary self-isolation vs. active syndromic surveillance and compulsory isolation. (B) Use of masks and respirators. (C) Improved social distancing among HCPs. (D) Increased physical separation of dialysis stations. (E) Patient isolation and preemptive isolation of exposed HCPs. (F) Combinations of inexpensive NPIs. Fig D. Attack rates for individual NPIs and selected NPIs in combination in Scenario 1 (infection source: HCP), R0 = 2.0, exp/exp (60%). (A) Voluntary self-isolation vs. active syndromic surveillance and compulsory isolation. (B) Use of masks and respirators. (C) Improved social distancing among HCPs. (D) Increased physical separation of dialysis stations. (E) Patient isolation and preemptive isolation of exposed HCPs. (F) Combinations of inexpensive NPIs. Fig E. Cumulative distributions of transmission events over 30 days in Baseline simulation on R0 = 2.5, and the exp/exp (60%). (A) Scenario 1: dialysis patient is the infection source. (B) Scenario 2: HC [file pcbi.1009177.s001.pdf]

Contents

1 S1 Appendix **1**
1.1 Simulation results on *exp/exp (60%)* shedding model 2
1.1.1 $R_0 = 2.0$, $\gamma = 2.0$ 2
1.1.2 $R_0 = 2.5$, $\gamma = 2.0$ 5
1.1.3 $R_0 = 3.0$, $\gamma = 1.5$ 8
1.1.4 $R_0 = 3.0$, $\gamma = 2.5$ 12
1.2 Simulation results on *exp/exp (20%)* shedding model 16
1.2.1 $R_0 = 3.0$, $\gamma = 2.0$ 16
1.3 Additional analyses on voluntary self-isolation 19
1.4 Simulation results on the other days of data 20
1.4.1 Day 2 20
1.4.2 Day 6 24
1.4.3 Day 7 28
1.4.4 Day 8 32
1.4.5 Day 9 36

1 S1 Appendix

In the main paper, we showed simulation results focusing on the worst case modeling assumptions (i.e., $R_0 = 3.0$, and the *exp/exp (60%)* shedding model). Here, in the Appendix, we show results on other settings of the parameters as follows:

- *exp/exp (60%)* shedding model, $R_0 \in \{2.0, 2.5\}$
- *exp/exp (60%)* shedding model, $R_0 = 3.0$, $\gamma \in \{1.5, 2.5\}$
- *exp/exp (20%)* shedding model, $R_0 = 3.0$
- Additional analyses on voluntary self-isolation
- *exp/exp (60%)* shedding model, $R_0 = 3.0$, on the other days of data

1.1 Simulation results on *exp/exp (60%)* shedding model

1.1.1 $R_0 = 2.0$, $\gamma = 2.0$

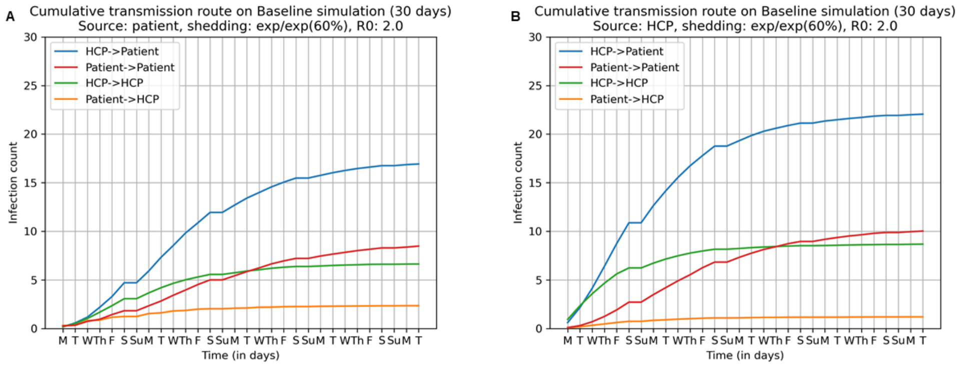

Fig A. Cumulative distributions of transmission events over 30 days in Baseline simulation on $R_0 = 2.0$, and the *exp/exp (60%)*. (A) Scenario 1: dialysis patient is the infection source. (B) Scenario 2: HCP is the infection source.

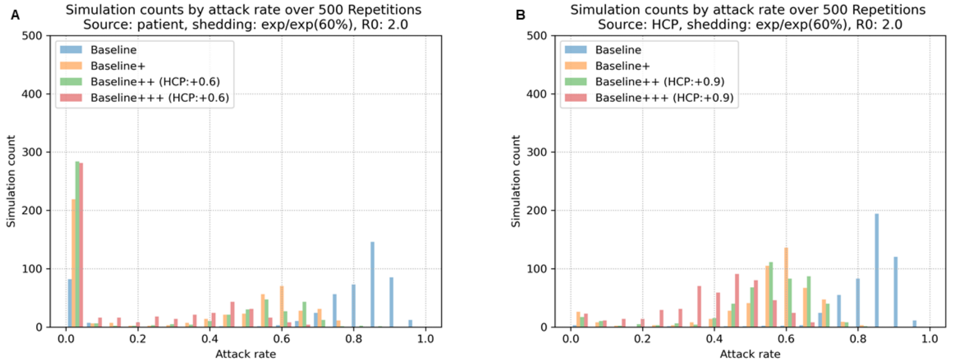

Fig B. Frequency of replicates as a function of attack rates for different NPIs on $R_0 = 2.0$, and the *exp/exp (60%)*. (A) Scenario 1. (B) Scenario 2.

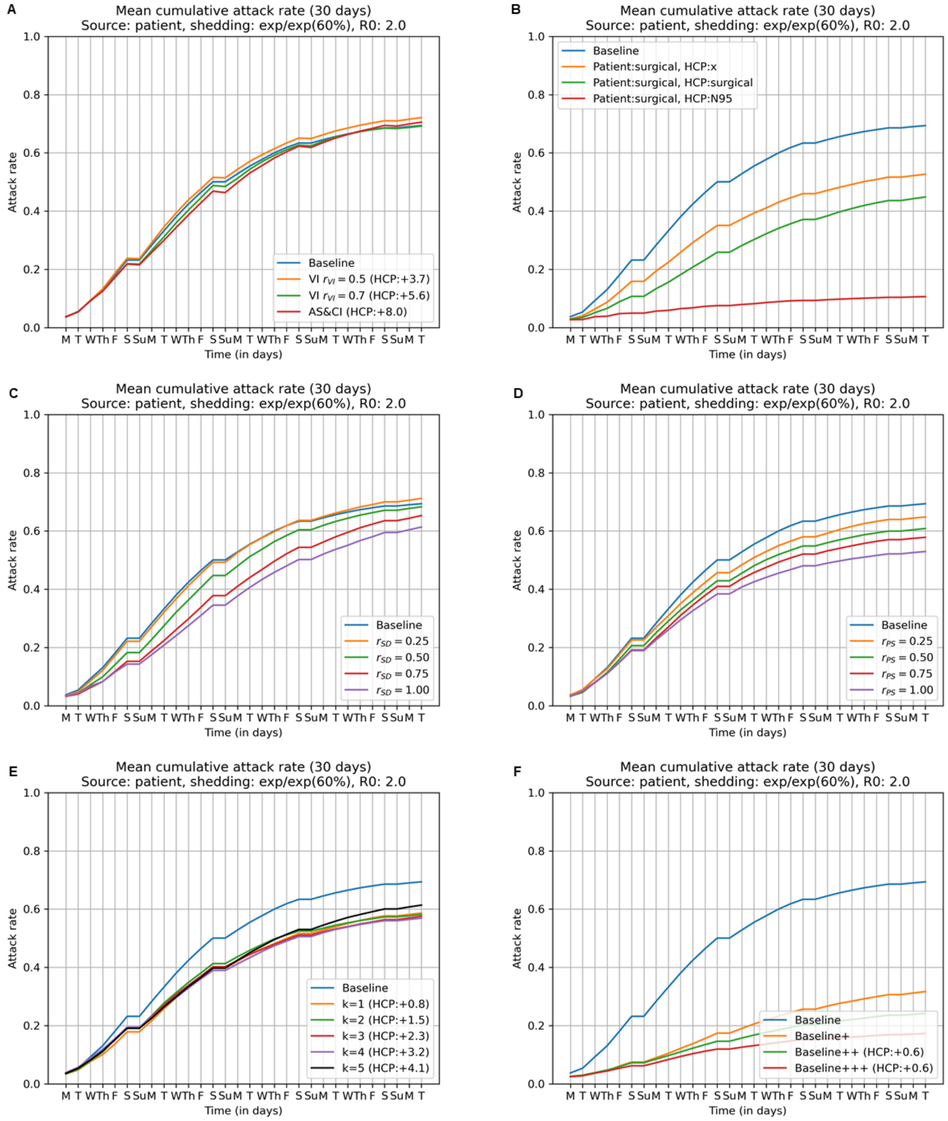

Fig C. Attack rates for individual NPIs and selected NPIs in combination in Scenario 1 (infection source: dialysis patient), $R_0 = 2.0$, *exp/exp (60%)*. (A) Voluntary self-isolation vs. active syndromic surveillance and compulsory isolation. (B) Use of masks and respirators. (C) Improved social distancing among HCPs. (D) Increased physical separation of dialysis stations. (E) Patient isolation and preemptive isolation of exposed HCPs. (F) Combinations of inexpensive NPIs.

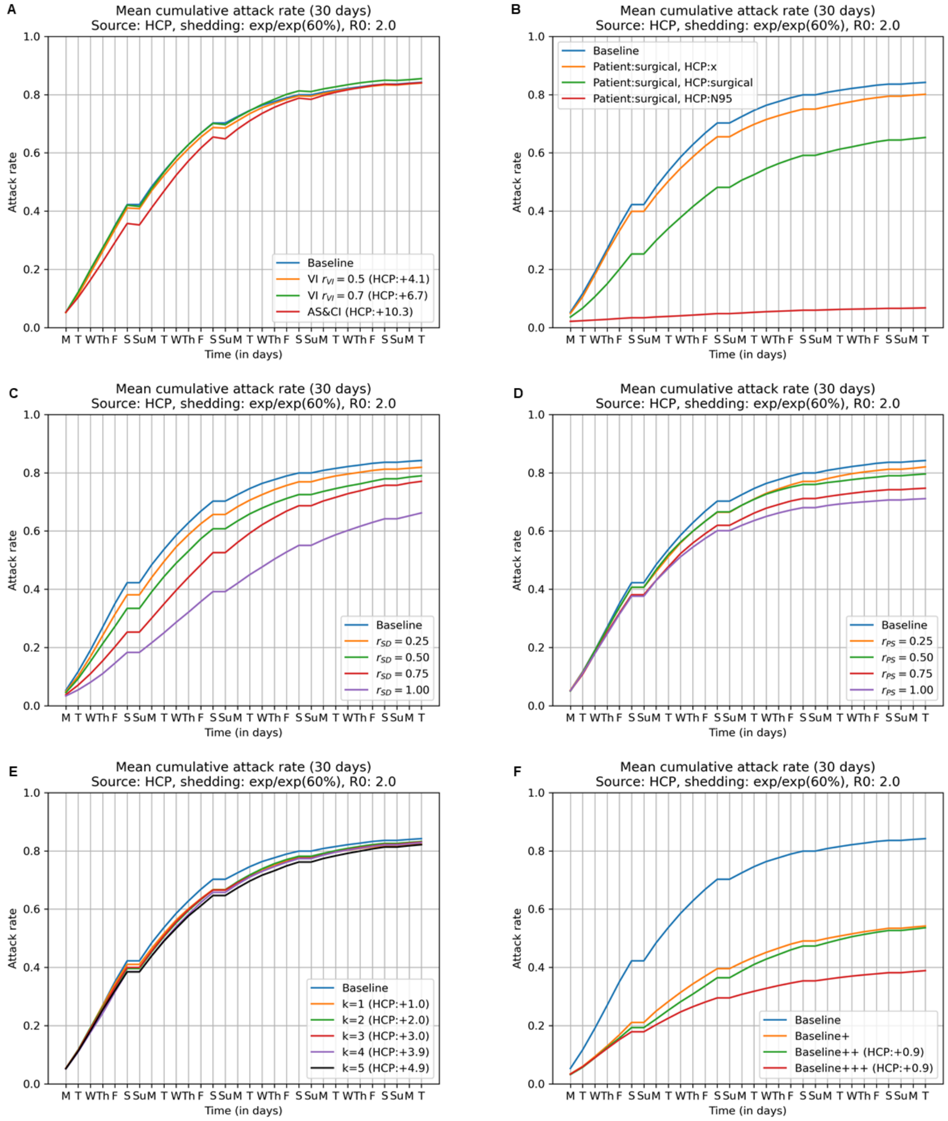

Fig D. Attack rates for individual NPIs and selected NPIs in combination in Scenario 1 (infection source: HCP), $R_0 = 2.0$, *exp/exp (60%).* (A) Voluntary self-isolation vs. active syndromic surveillance and compulsory isolation. (B) Use of masks and respirators. (C) Improved social distancing among HCPs. (D) Increased physical separation of dialysis stations. (E) Patient isolation and preemptive isolation of exposed HCPs. (F) Combinations of inexpensive NPIs.

1.1.2 $R_0 = 2.5$, $\gamma = 2.0$

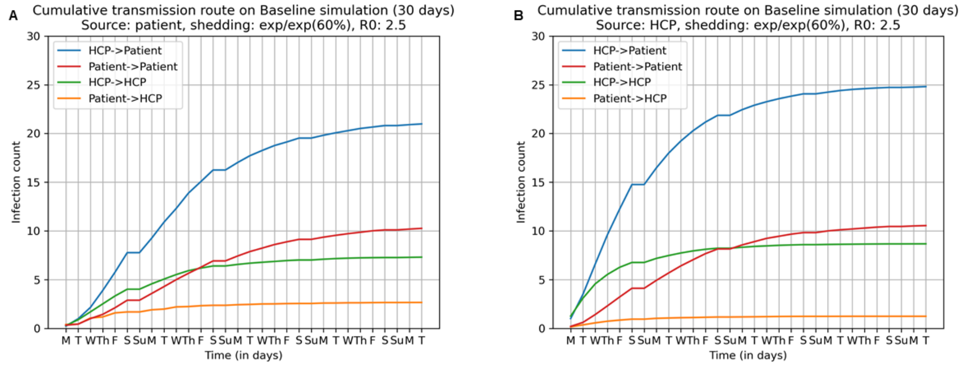

Fig E. Cumulative distributions of transmission events over 30 days in Baseline simulation on $R_0 = 2.5$, and the *exp/exp (60%)*. (A) Scenario 1: dialysis patient is the infection source. (B) Scenario 2: HCP is the infection source.

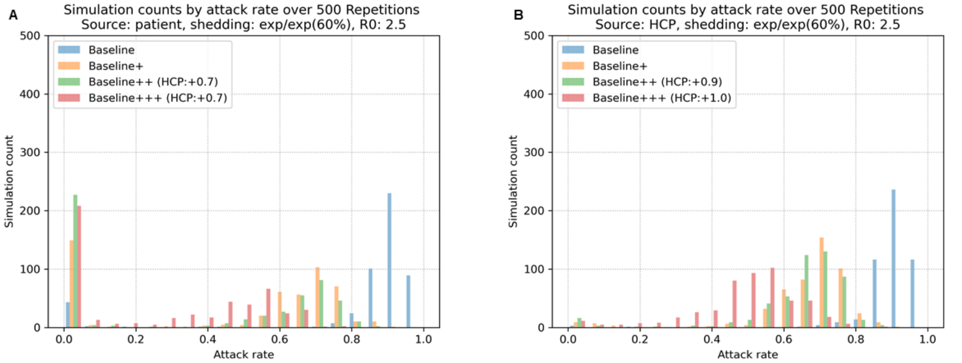

Fig F. Frequency of replicates as a function of attack rates for different NPIs on $R_0 = 2.5$, and the *exp/exp (60%)*. (A) Scenario 1. (B) Scenario 2.

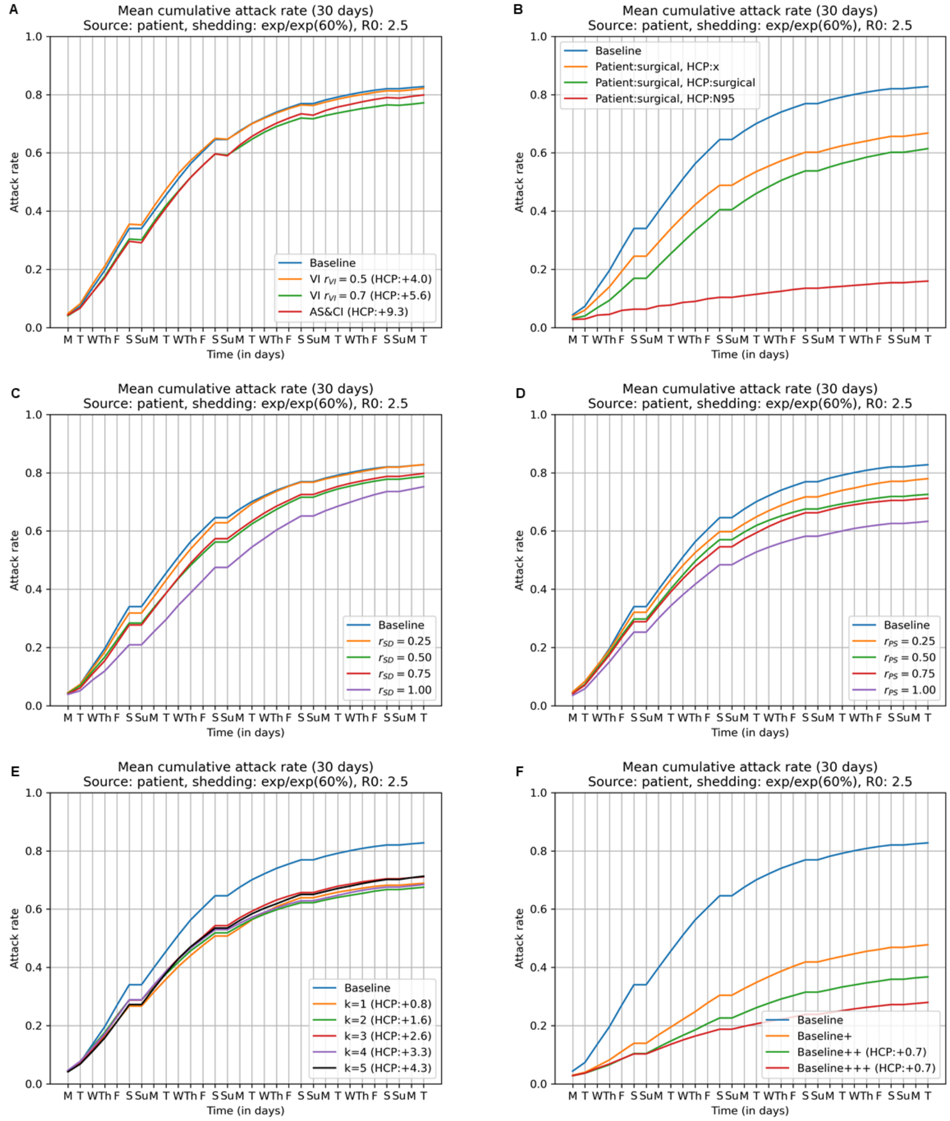

Fig G. Attack rates for individual NPIs and selected NPIs in combination in Scenario 1 (infection source: dialysis patient), $R_0 = 2.5$, *exp/exp (60%)*. (A) Voluntary self-isolation vs. active syndromic surveillance and compulsory isolation. (B) Use of masks and respirators. (C) Improved social distancing among HCPs. (D) Increased physical separation of dialysis stations. (E) Patient isolation and preemptive isolation of exposed HCPs. (F) Combinations of inexpensive NPIs.

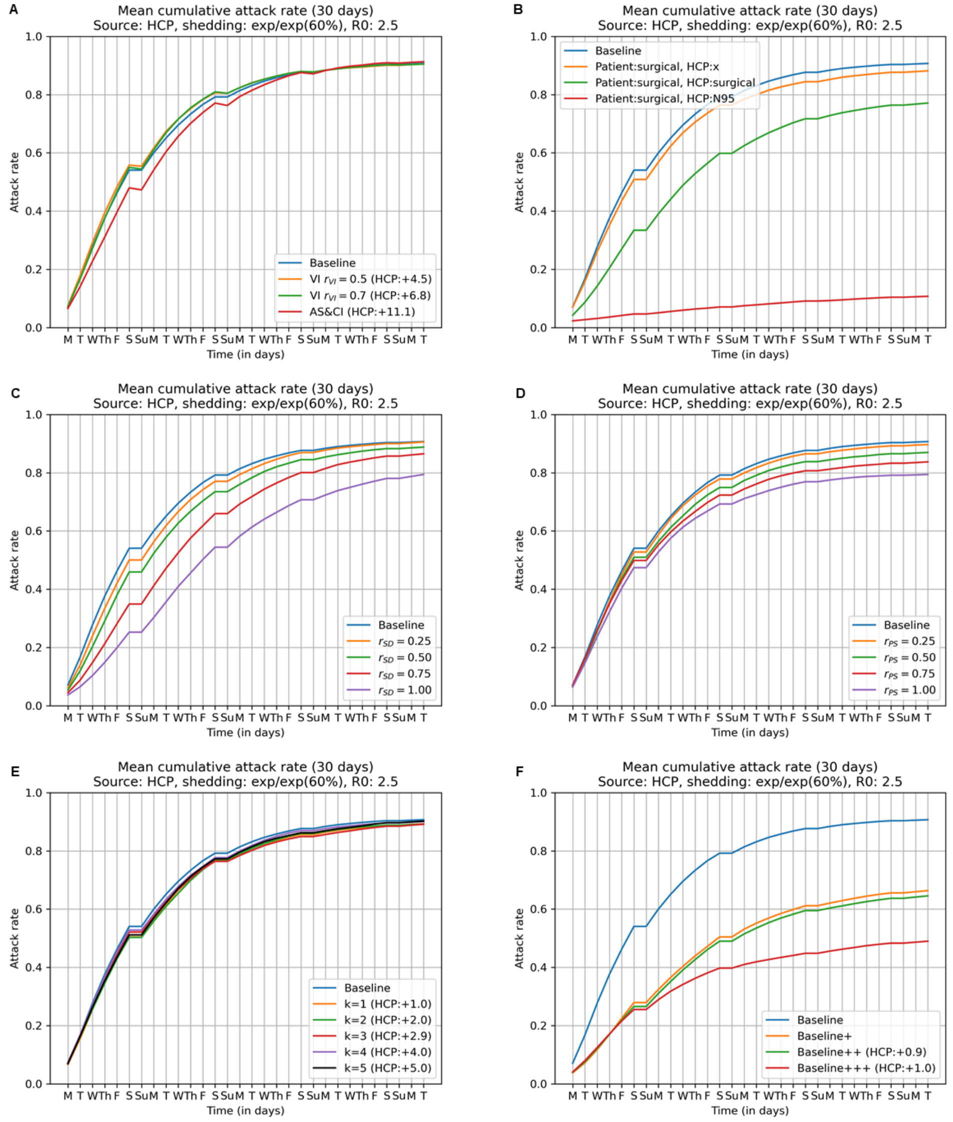

Fig H. Attack rates for individual NPIs and selected NPIs in combination in Scenario 1 (infection source: HCP), $R_0 = 2.5$, *exp/exp (60%)*. (A) Voluntary self-isolation vs. active syndromic surveillance and compulsory isolation. (B) Use of masks and respirators. (C) Improved social distancing among HCPs. (D) Increased physical separation of dialysis stations. (E) Patient isolation and preemptive isolation of exposed HCPs. (F) Combinations of inexpensive NPIs.

1.1.3 $R_0 = 3.0$, $\gamma = 1.5$

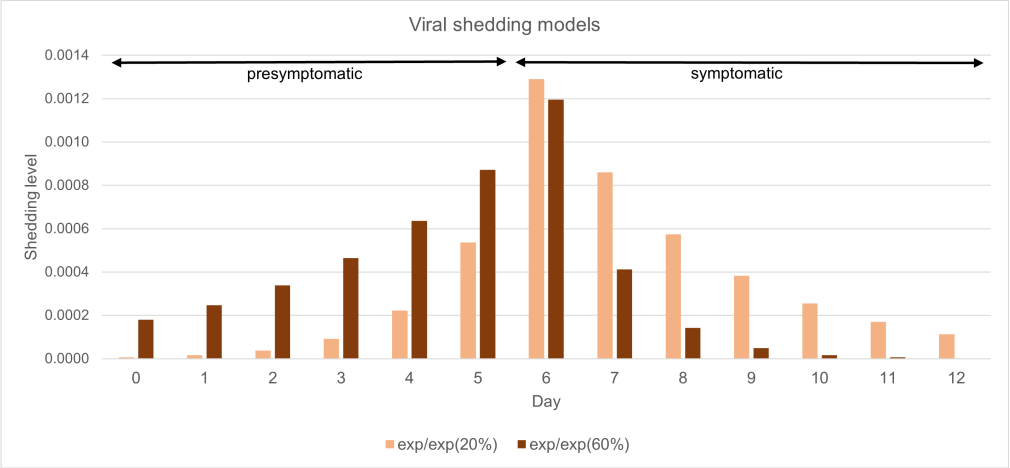

Fig I. Viral shedding models. The bar charts show the shedding level of an infected individual whose presymptomatic period and symptomatic period are 6 and 7 days, respectively. The percentage in the parenthesis of each model corresponds to the percentage of shedding during the presymptomatic state (P) relative to the total shedding volume. The peak shedding level differs in the two models, but the total shedding volume remains the same. The shedding models are calibrated by setting the shedding ramp down parameter of the *exp/exp (20%)* shedding model $\gamma = 1.5$.

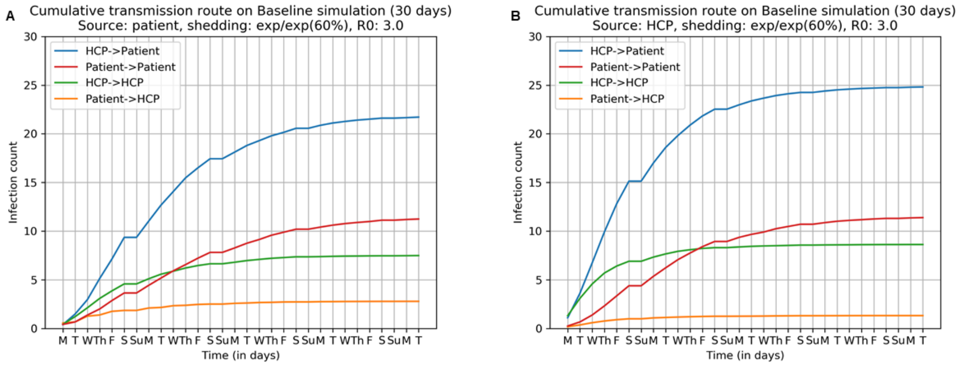

Fig J. Cumulative distributions of transmission events over 30 days in Baseline simulation on $R_0 = 3.0$, and the *exp/exp (60%)* shedding model where $\gamma = 1.5$. (A) Scenario 1: dialysis patient is the infection source. (B) Scenario 2: HCP is the infection source.

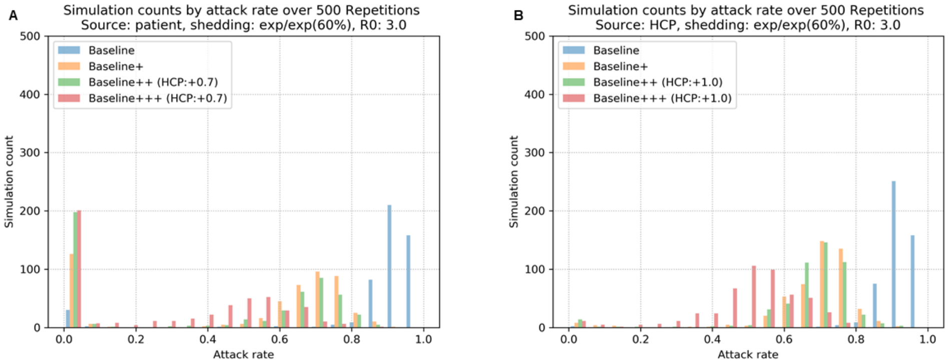

Fig K. Frequency of replicates as a function of attack rates for different NPIs on $R_0 = 3.0$, and the *exp/exp (60%)* shedding model where $\gamma = 1.5$. (A) Scenario 1. (B) Scenario 2.

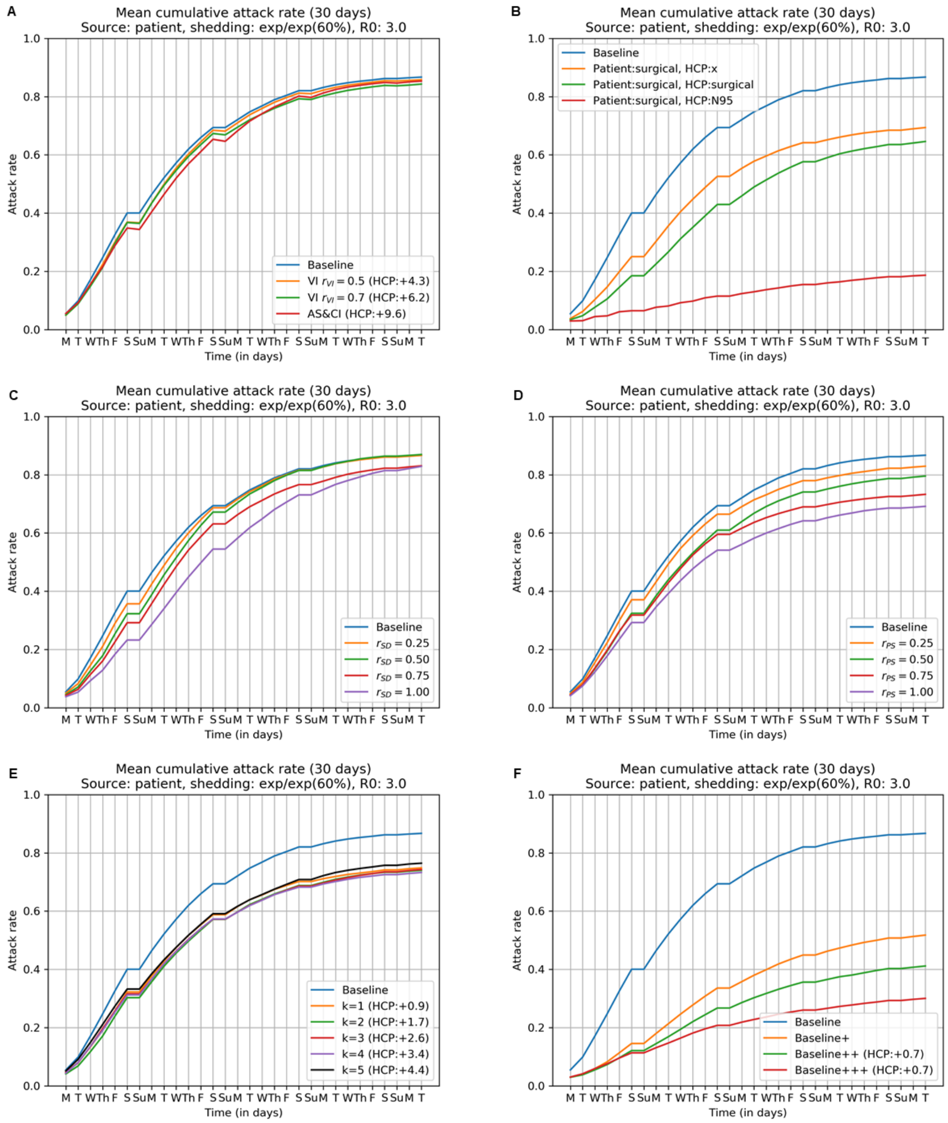

Fig L. Attack rates for individual NPIs and selected NPIs in combination in Scenario 1 (infection source: dialysis patient), $R_0 = 3.0$, *exp/exp (60%)*, where $\gamma = 1.5$. (A) Voluntary self-isolation vs. active syndromic surveillance and compulsory isolation. (B) Use of masks and respirators. (C) Improved social distancing among HCPs. (D) Increased physical separation of dialysis stations. (E) Patient isolation and preemptive isolation of exposed HCPs. (F) Combinations of inexpensive NPIs.

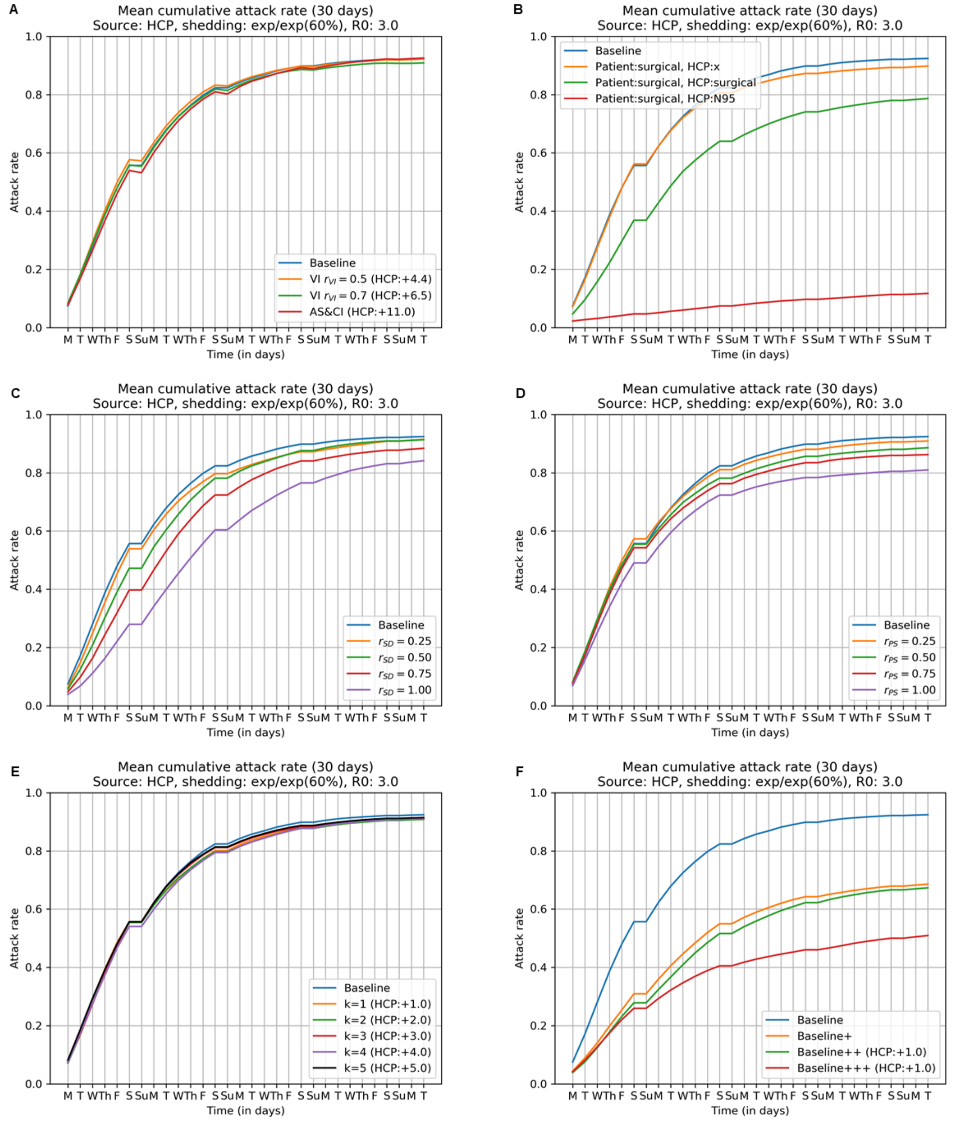

Fig M. Attack rates for individual NPIs and selected NPIs in combination in Scenario 1 (infection source: HCP), $R_0 = 3.0$, *exp/exp (60%)*, where $\gamma = 1.5$. (A) Voluntary self-isolation vs. active syndromic surveillance and compulsory isolation. (B) Use of masks and respirators. (C) Improved social distancing among HCPs. (D) Increased physical separation of dialysis stations. (E) Patient isolation and preemptive isolation of exposed HCPs. (F) Combinations of inexpensive NPIs.

1.1.4 $R_0 = 3.0$, $\gamma = 2.5$

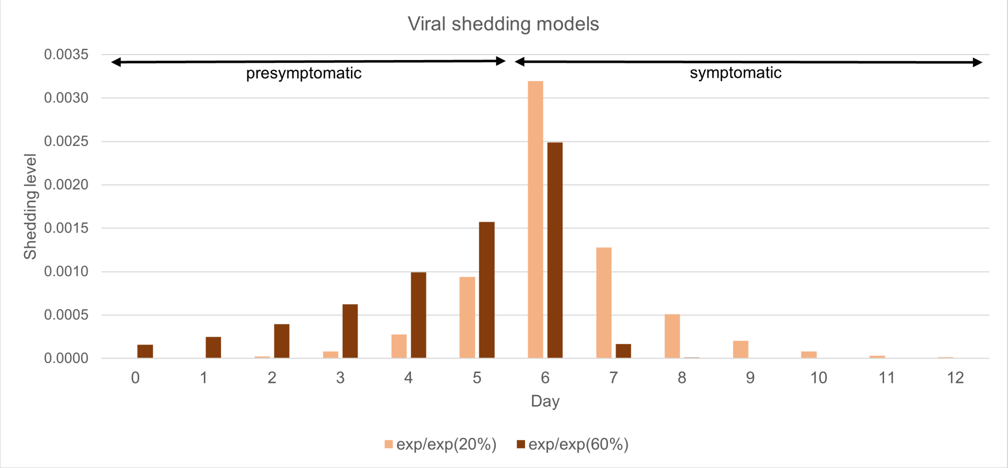

Fig N. Viral shedding models. The bar charts show the shedding level of an infected individual whose presymptomatic period and symptomatic period are 6 and 7 days, respectively. The percentage in the parenthesis of each model corresponds to the percentage of shedding during the presymptomatic state (P) relative to the total shedding volume. The peak shedding level differs in the two models, but the total shedding volume remains the same. The shedding models are calibrated by setting the shedding ramp down parameter of the *exp/exp (20%)* shedding model $\gamma = 2.5$.

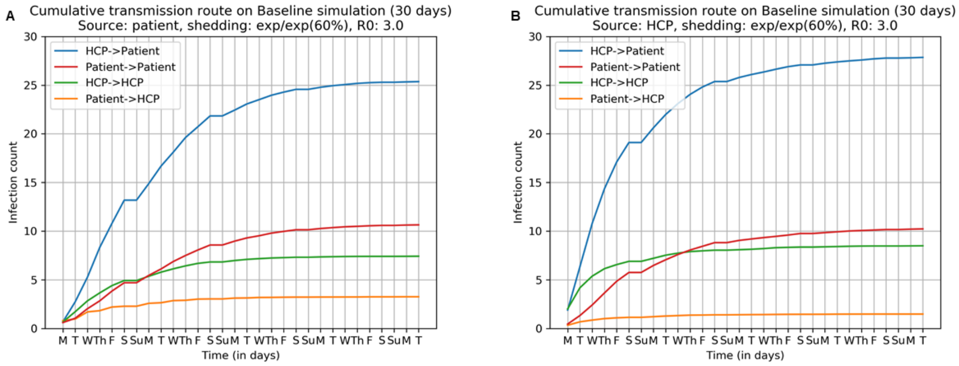

Fig O. Cumulative distributions of transmission events over 30 days in Baseline simulation on $R_0 = 3.0$, and the *exp/exp (60%)* shedding model where $\gamma = 2.5$. (A) Scenario 1: dialysis patient is the infection source. (B) Scenario 2: HCP is the infection source.

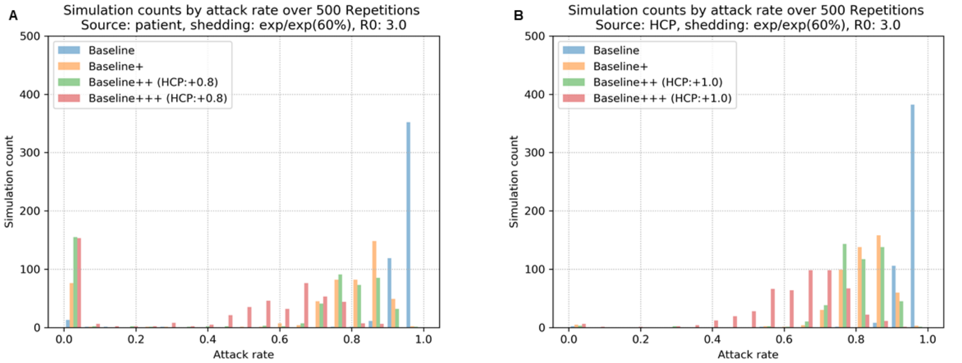

Fig P. Frequency of replicates as a function of attack rates for different NPIs on $R_0 = 3.0$, and the *exp/exp (60%)* shedding model where $\gamma = 2.5$. (A) Scenario 1. (B) Scenario 2.

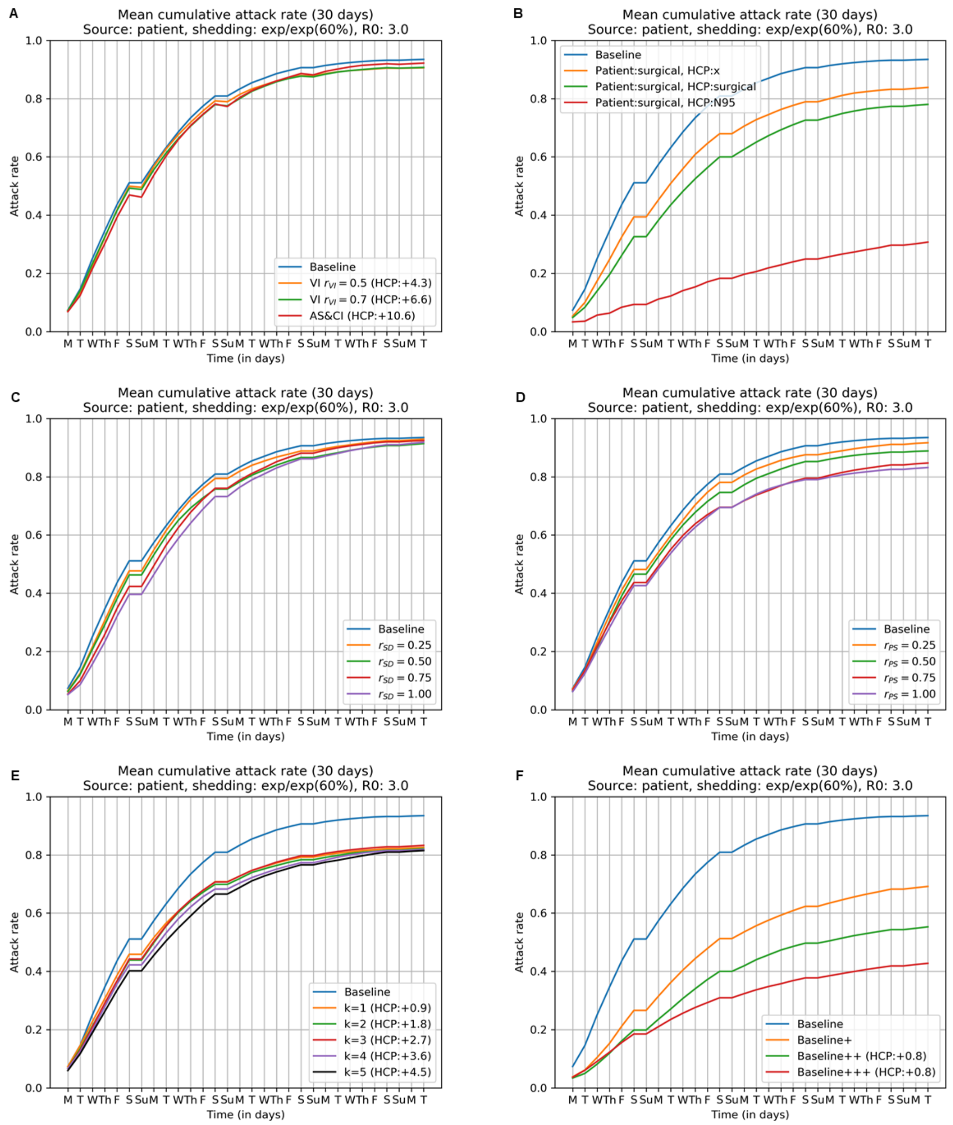

Fig Q. Attack rates for individual NPIs and selected NPIs in combination in Scenario 1 (infection source: dialysis patient), $R_0 = 3.0$, ***exp/exp (60%)*****, where** $\gamma = 2.5$**.** (A) Voluntary self-isolation vs. active syndromic surveillance and compulsory isolation. (B) Use of masks and respirators. (C) Improved social distancing among HCPs. (D) Increased physical separation of dialysis stations. (E) Patient isolation and preemptive isolation of exposed HCPs. (F) Combinations of inexpensive NPIs.

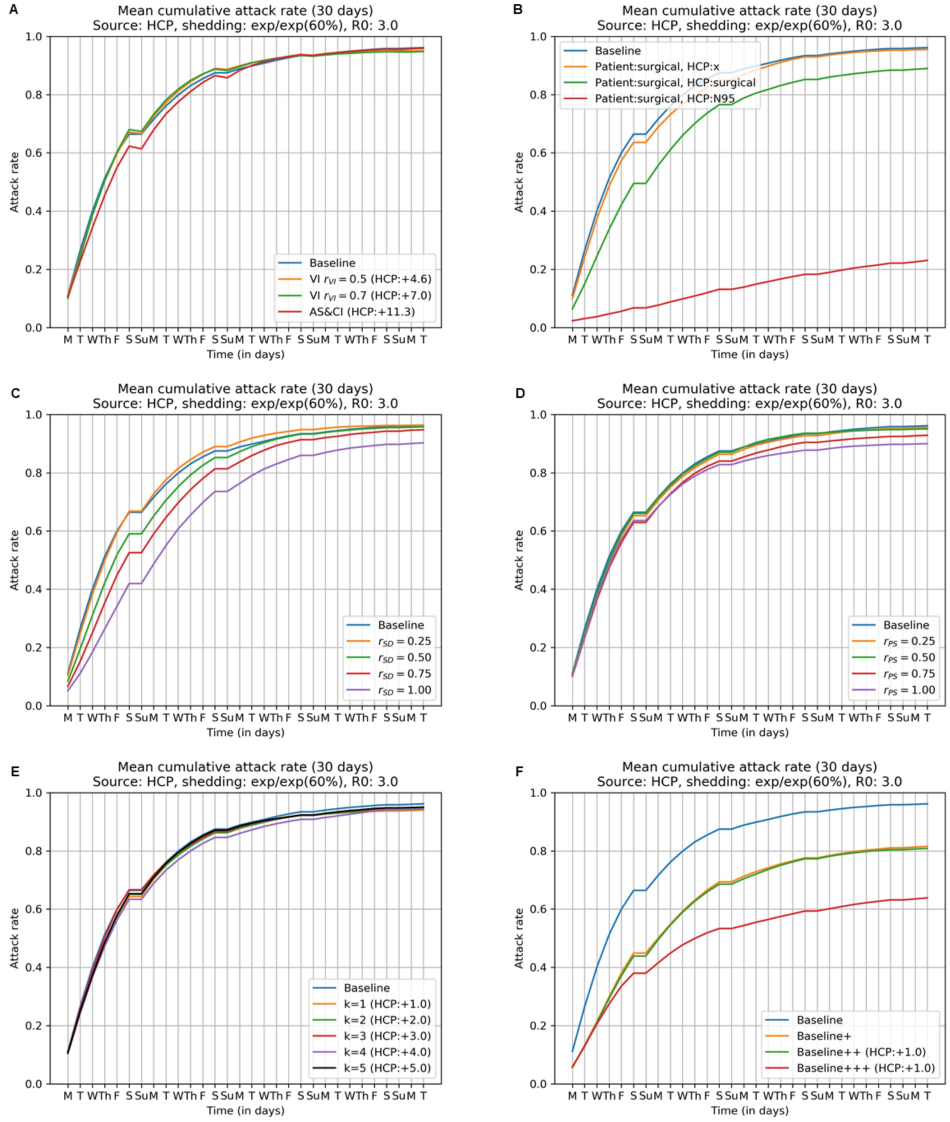

Fig R. Attack rates for individual NPIs and selected NPIs in combination in Scenario 1 (infection source: HCP), $R_0 = 3.0$, *exp/exp (60%)*, where $\gamma = 2.5$. (A) Voluntary self-isolation vs. active syndromic surveillance and compulsory isolation. (B) Use of masks and respirators. (C) Improved social distancing among HCPs. (D) Increased physical separation of dialysis stations. (E) Patient isolation and preemptive isolation of exposed HCPs. (F) Combinations of inexpensive NPIs.

1.2 Simulation results on *exp/exp (20%)* shedding model

1.2.1 $R_0 = 3.0$, $\gamma = 2.0$

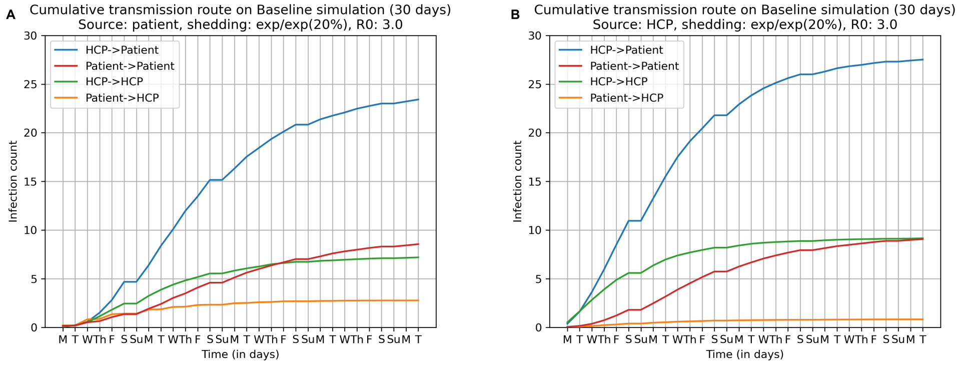

Fig S. Cumulative distributions of transmission events over 30 days in Baseline simulation on $R_0 = 3.0$, and the *exp/exp (20%)* shedding model. (A) Scenario 1: dialysis patient is the infection source. (B) Scenario 2: HCP is the infection source.

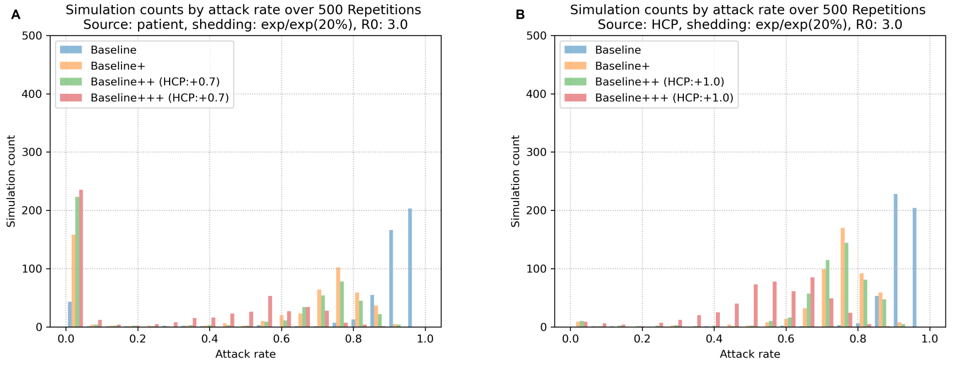

Fig T. Frequency of replicates as a function of attack rates for different NPIs on $R_0 = 3.0$, and the *exp/exp (20%)* shedding model. (A) Scenario 1. (B) Scenario 2.

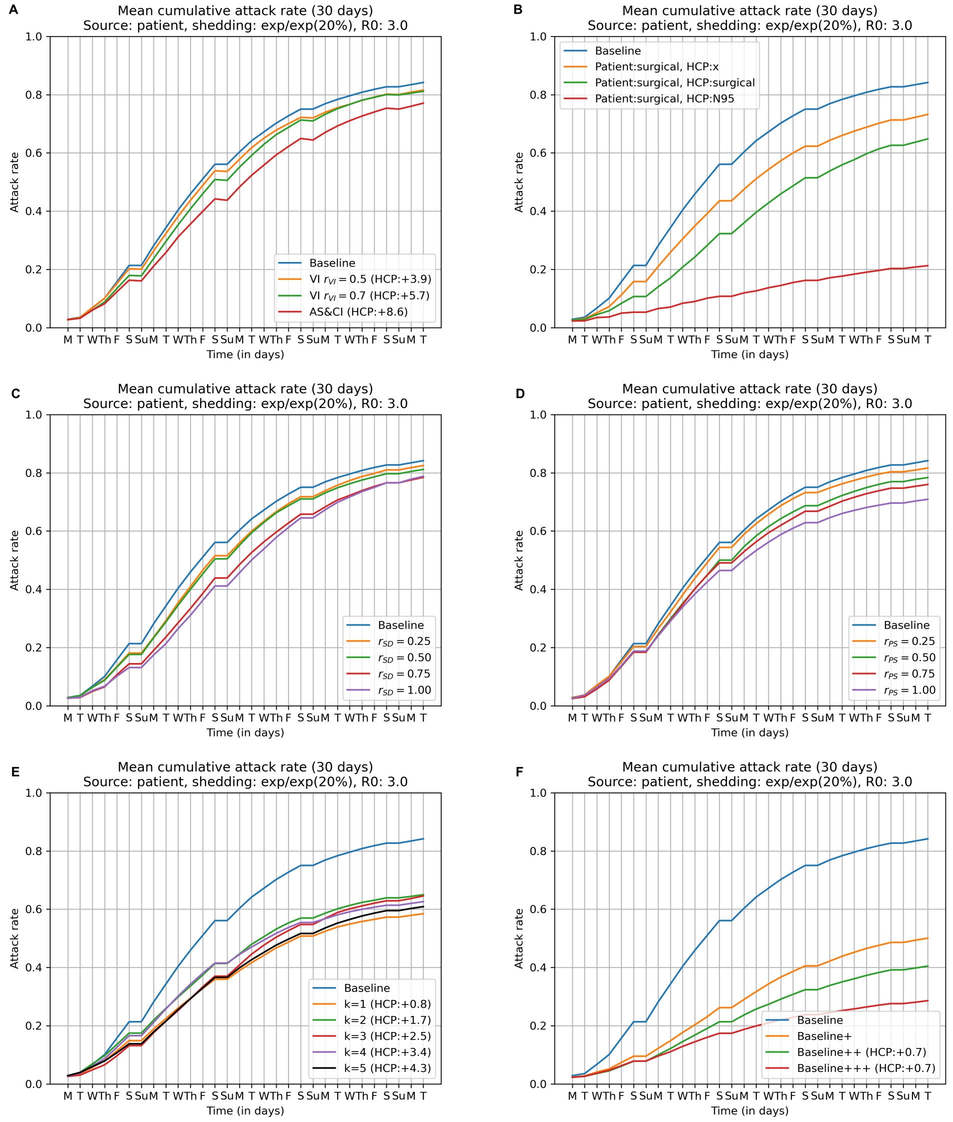

Fig U. Attack rates for individual NPIs and selected NPIs in combination in Scenario 1 (infection source: dialysis patient), $R_0 = 3.0$, *exp/exp (20%)*. (A) Voluntary self-isolation vs. active syndromic surveillance and compulsory isolation. (B) Use of masks and respirators. (C) Improved social distancing among HCPs. (D) Increased physical separation of dialysis stations. (E) Patient isolation and preemptive isolation of exposed HCPs. (F) Combinations of inexpensive NPIs.

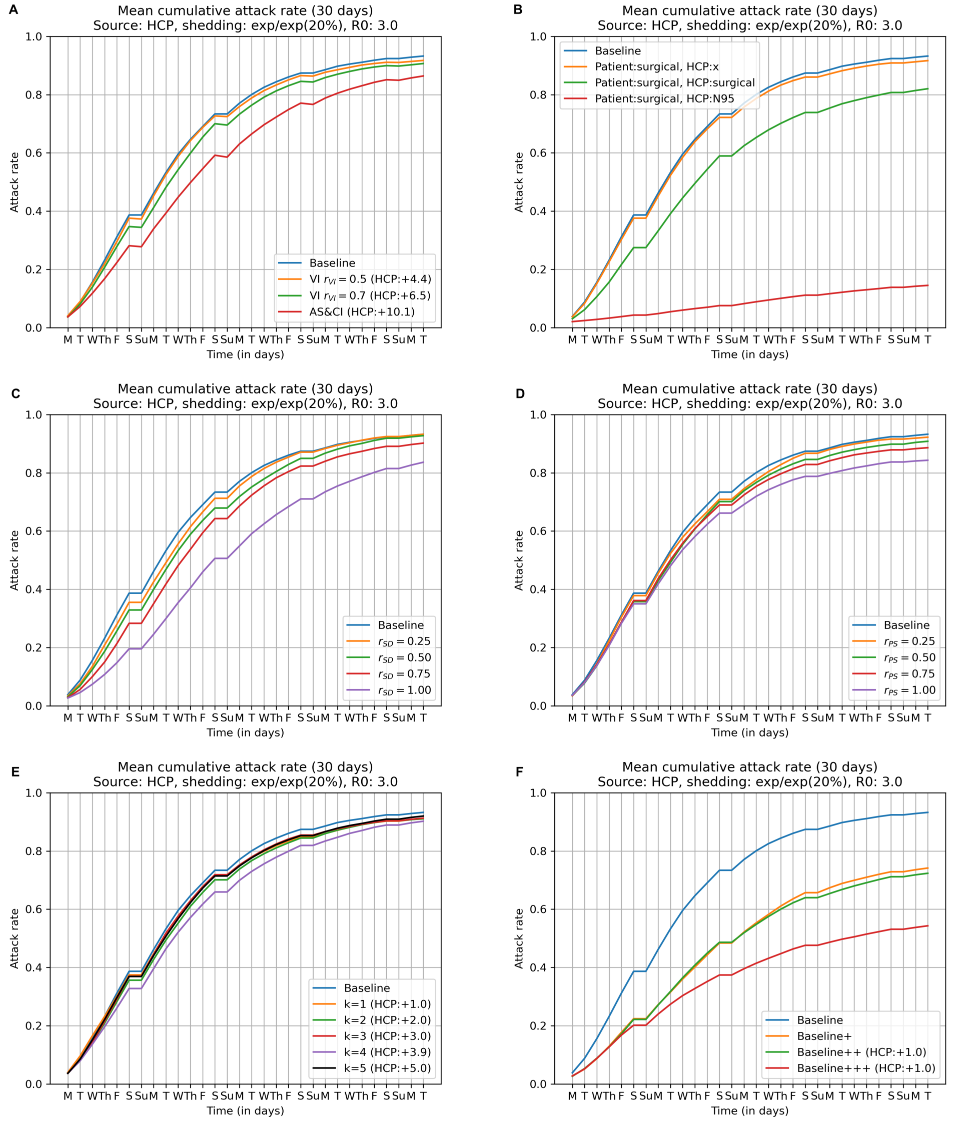

Fig V. Attack rates for individual NPIs and selected NPIs in combination in Scenario 1 (infection source: HCP), $R_0 = 3.0$, *exp/exp (20%)*. (A) Voluntary self-isolation vs. active syndromic surveillance and compulsory isolation. (B) Use of masks and respirators. (C) Improved social distancing among HCPs. (D) Increased physical separation of dialysis stations. (E) Patient isolation and preemptive isolation of exposed HCPs. (F) Combinations of inexpensive NPIs.

1.3 Additional analyses on voluntary self-isolation

While there have been studies on "presenteeism" among healthcare workers, as far as we know, there are no studies on HCP presenteeism with COVID-19 symptoms. Specifically, there are no studies from which we can infer values of the voluntary isolation rate parameter r_{VI}. In the main paper, we picked a range for this parameter that seemed reasonable that is $r_{VI} \in \{0.5, 0.7\}$. We also explored a wider range for this parameter, namely {0.4, 0.5, 0.6, 0.7, 0.8}. We observed a very small difference in the attack rates using this expanded range of parameter values. In Table A, we report the cumulative attack rates for the voluntary self-isolation for these parameters under different modeling assumptions. Notice that the attack rates do not change much for different choices of voluntary isolation rates. More importantly, as discussed in the paper, voluntary isolation was not considered an effective intervention and was not included in the set of effective interventions, e.g., Baseline+, Baseline++, nor Baseline+++.

Table A. Attack rates for voluntary isolation compliance rate $r_{VI} \in$ {0.4, 0.5, 0.6, 0.7, 0.8} under different modeling assumptions. "SM" is short for "shedding models", "B" is short for "Baseline," in Scenario 1 the infection source is a dialysis patient and in Scenario 2 the infection source is a HCP.

SM	R0	Scenario 1						Scenario 2					
		B	Voluntary self-isolation					B	Voluntary self-isolation				
		-	0.4	0.5	0.6	0.7	0.8	-	0.4	0.5	0.6	0.7	0.8
20%	2.0	62%	66%	60%	62%	61%	60%	82%	80%	80%	82%	79%	79%
	2.5	75%	72%	74%	73%	74%	70%	88%	87%	87%	88%	87%	86%
	3.0	84%	80%	83%	80%	81%	79%	93%	93%	92%	91%	91%	91%
60%	2.0	69%	67%	71%	71%	69%	68%	84%	85%	85%	85%	85%	85%
	2.5	83%	81%	81%	80%	81%	80%	91%	91%	91%	90%	90%	90%
	3.0	88%	87%	87%	88%	86%	86%	95%	94%	93%	93%	93%	93%

1.4 Simulation results on the other days of data

We gathered ten days of HCP movement and interaction data where six days had 14.5-15 hours of observation (Day 2, Day 6, Day 7, Day 8, Day 9, and Day 10). The simulation results reported in the main paper used the interactions in Day 10. As a form of validation, we ran the same set of simulations using HCP movement and interaction patterns extracted from the other days data within our dataset. And while the attack rates varied slightly due to minor differences in the number of HCPs and patients as well as their underlying contact patterns, we observed the same relationship between intervention strategy and reduced attack rates as we moved from the Baseline strategy towards Baseline+++. Here, we show simulation results on *exp/exp (60%)* shedding model, $R_0 = 3.0$, and $\gamma = 2.0$ on the other days of data.

1.4.1 Day 2

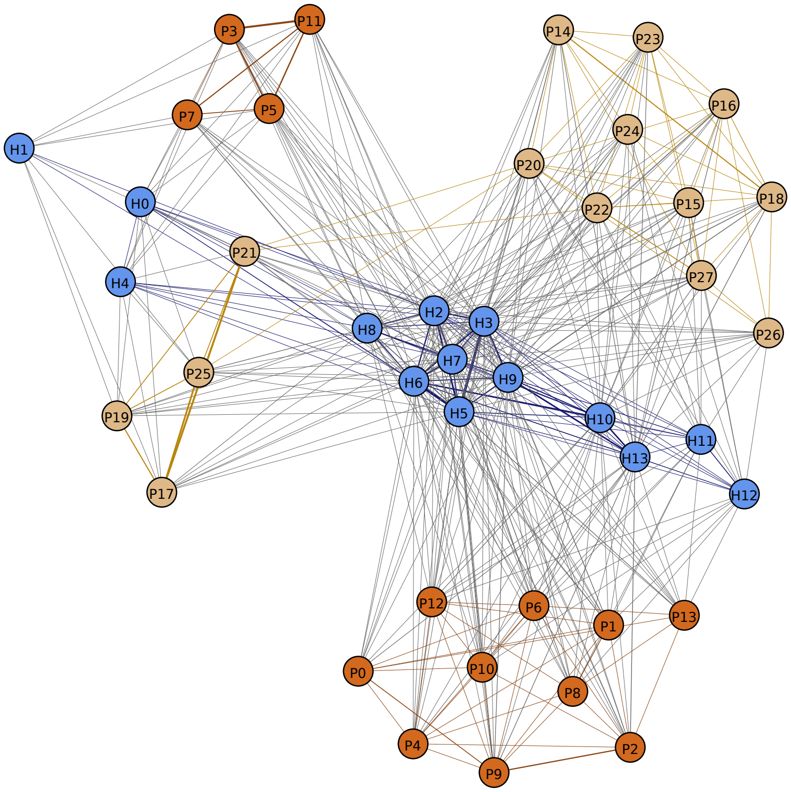

Fig W. HCP nodes, MWF patient nodes, and TThS patient nodes are depicted in blue, chocolate, and burlywood colors, respectively. We observe a total population of 42 agents (14 HCPs and 28 patients). Contacts within the same type of nodes are represented as edges colored according to node type. Grey colored edges represent HCP-patient contacts. The thickness of the edge corresponds to the contact duration.

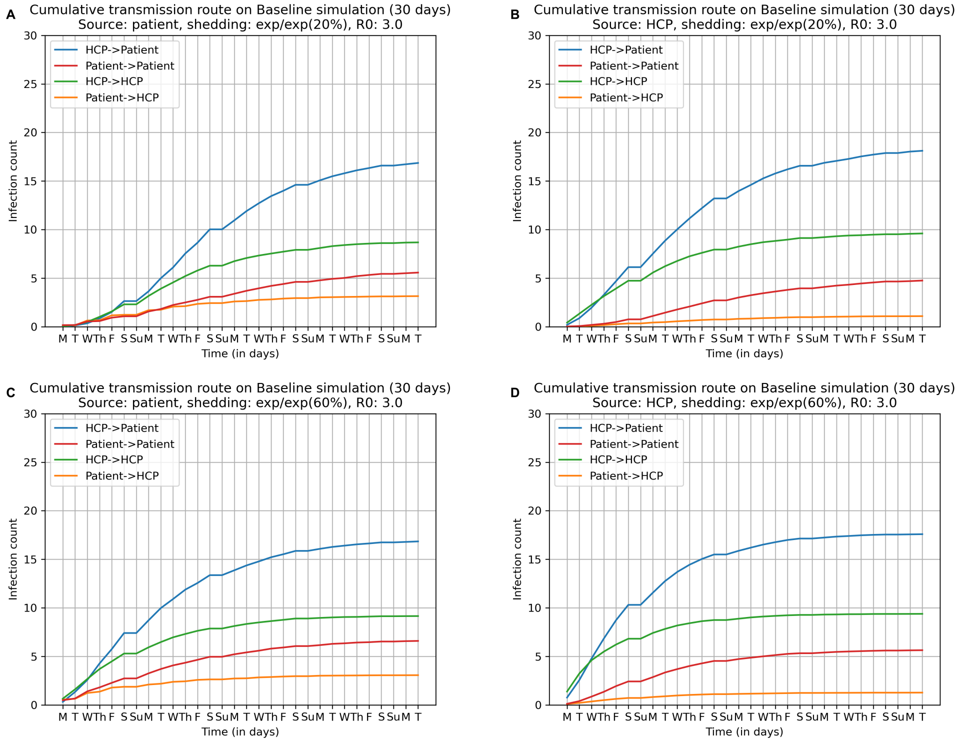

Fig X. Cumulative distributions of transmission events over 30 days in Baseline simulation on Day 2 and $R_0 = 3.0$. (A) the *exp/exp (20%)* shedding model on Scenario 1. (B) the *exp/exp (20%)* shedding model on Scenario 2. (C) the *exp/exp (60%)* shedding model on Scenario 1. (D) the *exp/exp (60%)* shedding model on Scenario 2.

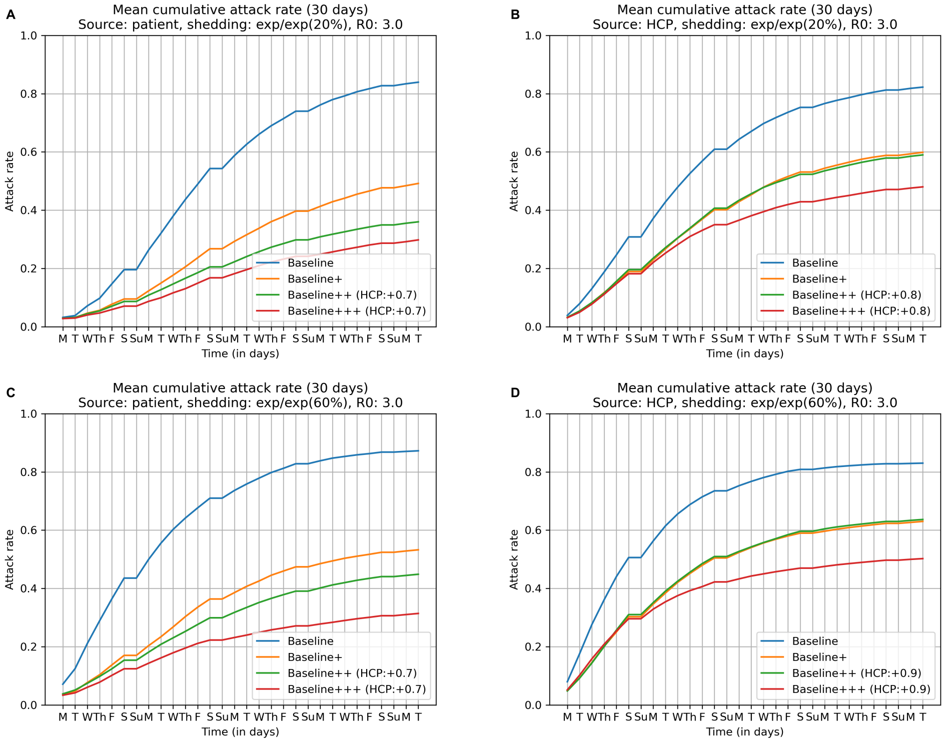

Fig Y. Attack rates for combinations of inexpensive NPIs on Day 2, $R_0 = 3.0$. (A) the *exp/exp (20%)* shedding model on Scenario 1. (B) the *exp/exp (20%)* shedding model on Scenario 2. (C) the *exp/exp (60%)* shedding model on Scenario 1. (D) the *exp/exp (60%)* shedding model on Scenario 2.

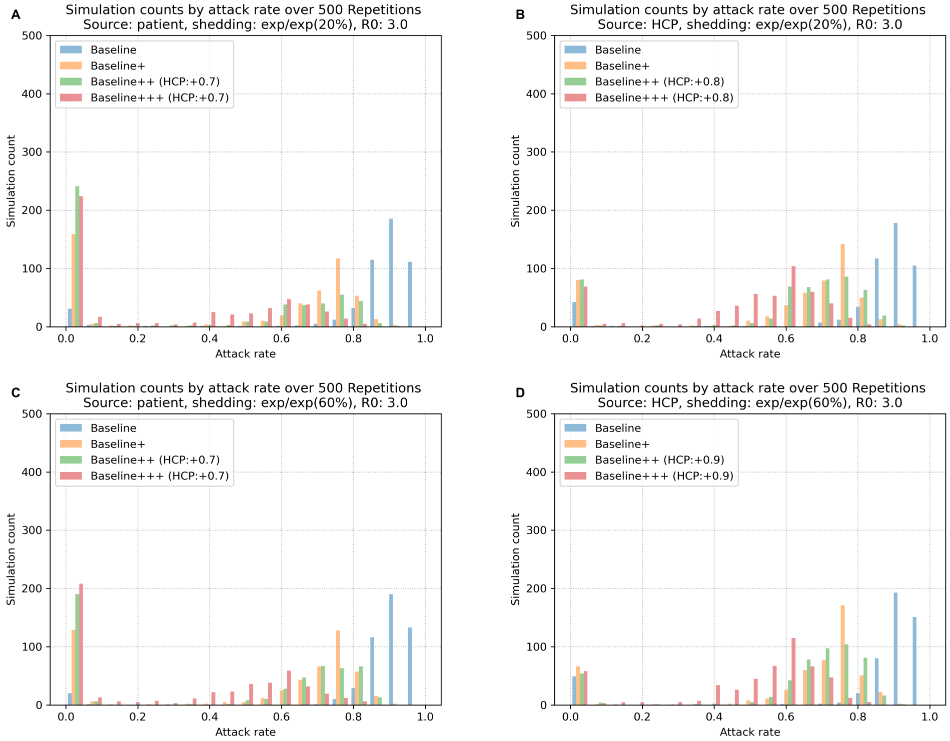

Fig Z. Frequency of replicates as a function of attack rates for combinations of inexpensive NPIs on Day 2, $R_0 = 3.0$. (A) the *exp/exp (20%)* shedding model on Scenario 1. (B) the *exp/exp (20%)* shedding model on Scenario 2. (C) the *exp/exp (60%)* shedding model on Scenario 1. (D) the *exp/exp (60%)* shedding model on Scenario 2.

1.4.2 Day 6

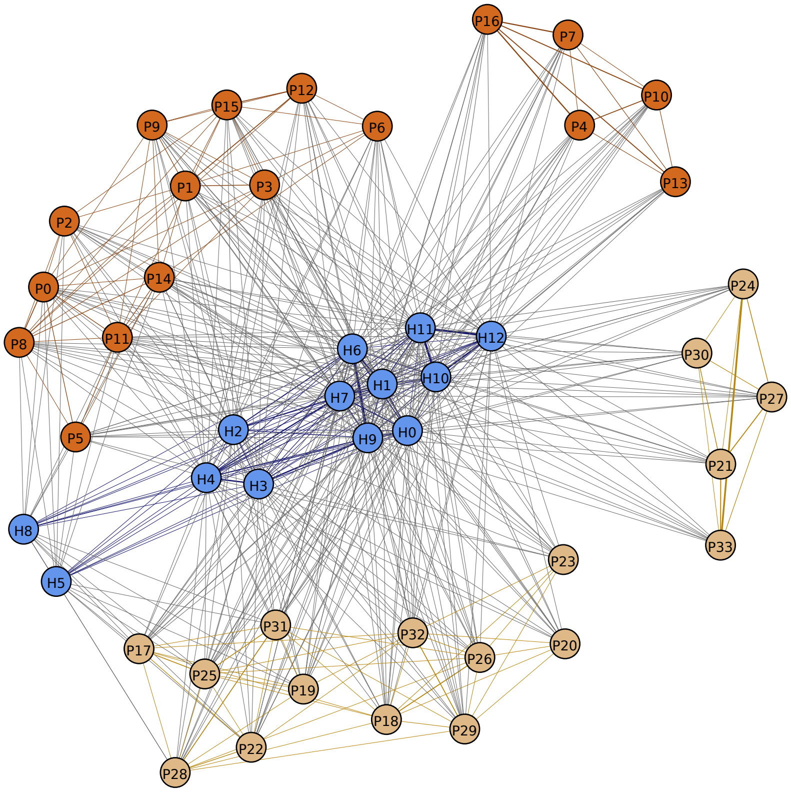

Fig AA. Contact network *G* on Day 6. We gathered ten days of HCP movement and interaction data where six days had 14.5-15 hours of observation (Day 2, Day 6, Day 7, Day 8, Day 9, and Day 10). HCP nodes, MWF patient nodes, and TThS patient nodes are depicted in blue, chocolate, and burlywood colors, respectively. We observe a total population of 47 agents (13 HCPs and 34 patients).

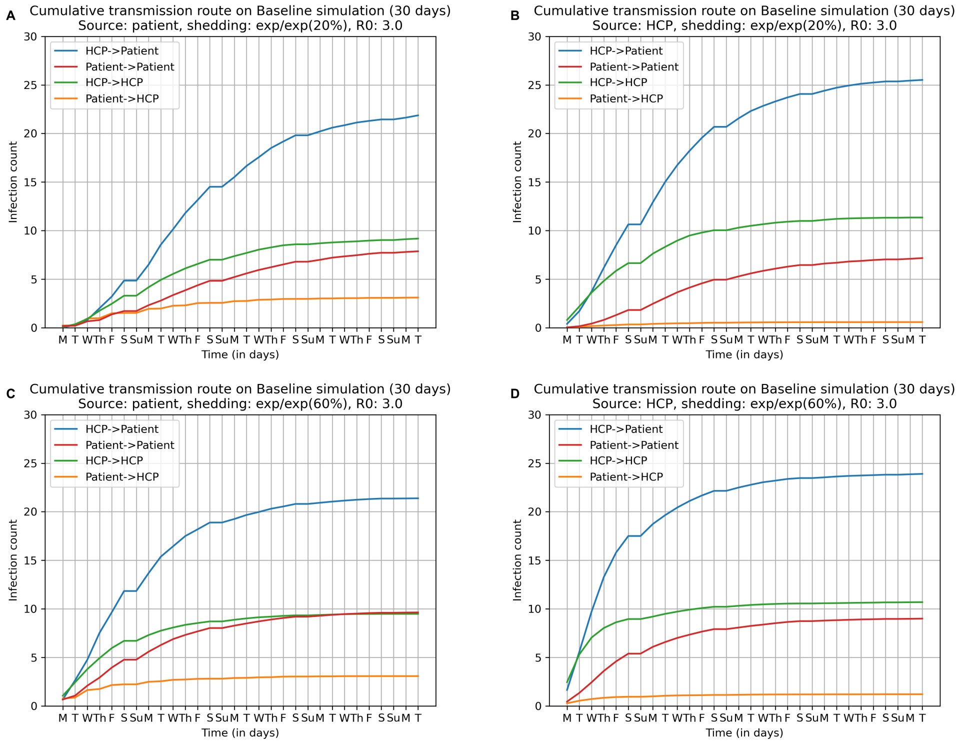

Fig AB. Cumulative distributions of transmission events over 30 days in Baseline simulation on Day 6 and $R_0 = 3.0$. (A) the *exp/exp (20%)* shedding model on Scenario 1. (B) the *exp/exp (20%)* shedding model on Scenario 2. (C) the *exp/exp (60%)* shedding model on Scenario 1. (D) the *exp/exp (60%)* shedding model on Scenario 2.

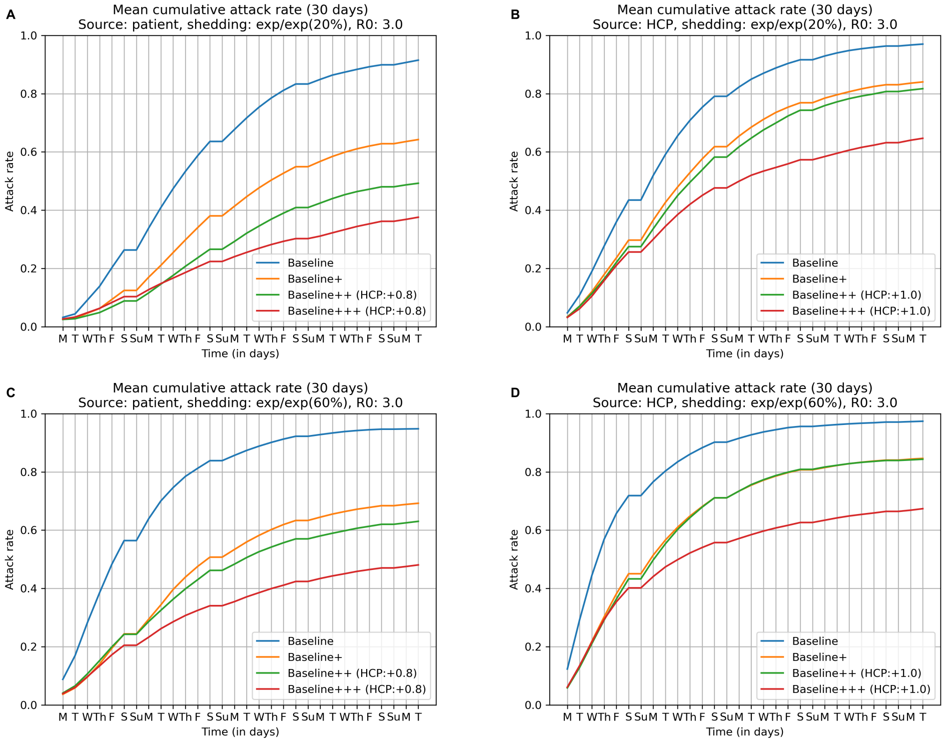

Fig AC. Attack rates for combinations of inexpensive NPIs on Day 6, $R_0 = 3.0$. (A) the *exp/exp (20%)* shedding model on Scenario 1. (B) the *exp/exp (20%)* shedding model on Scenario 2. (C) the *exp/exp (60%)* shedding model on Scenario 1. (D) the *exp/exp (60%)* shedding model on Scenario 2.

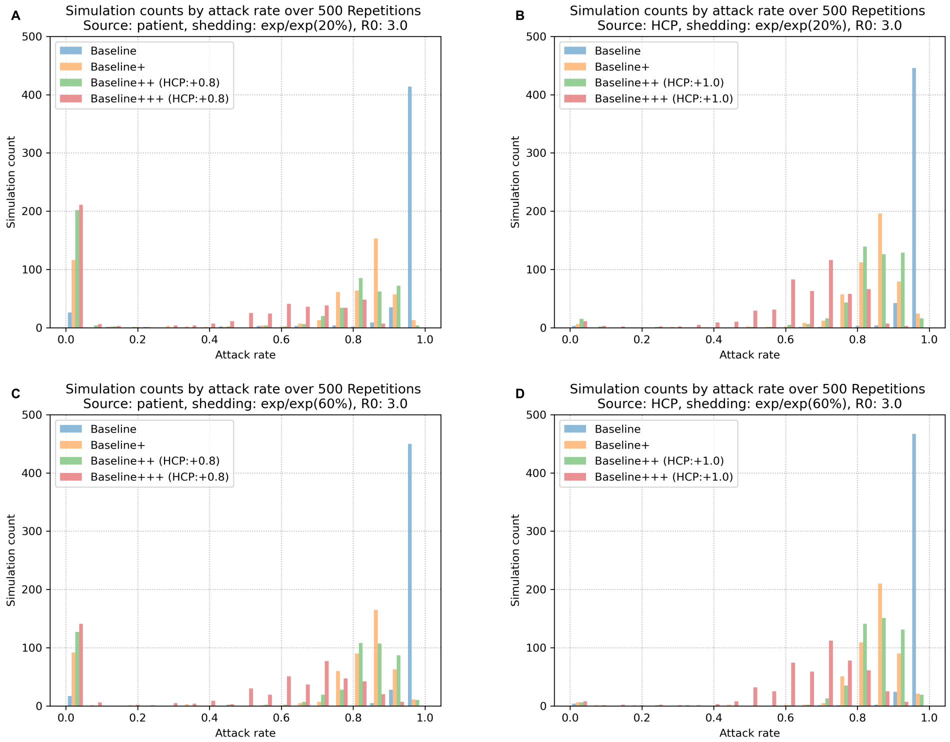

Fig AD. Frequency of replicates as a function of attack rates for combinations of inexpensive NPIs on Day 6, $R_0 = 3.0$. (A) the *exp/exp (20%)* shedding model on Scenario 1. (B) the *exp/exp (20%)* shedding model on Scenario 2. (C) the *exp/exp (60%)* shedding model on Scenario 1. (D) the *exp/exp (60%)* shedding model on Scenario 2.

1.4.3 Day 7

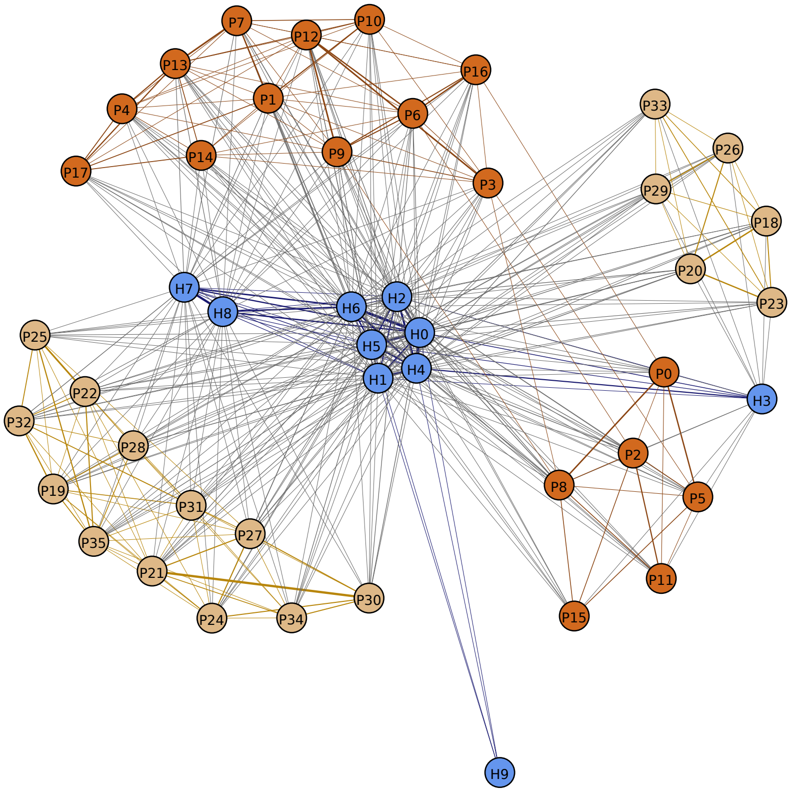

Fig AE. Contact network G **on Day 7.** We gathered ten days of HCP movement and interaction data where six days had 14.5-15 hours of observation (Day 2, Day 6, Day 7, Day 8, Day 9, and Day 10). HCP nodes, MWF patient nodes, and TThS patient nodes are depicted in blue, chocolate, and burlywood colors, respectively. We observe a total population of 46 agents (10 HCPs and 36 patients).

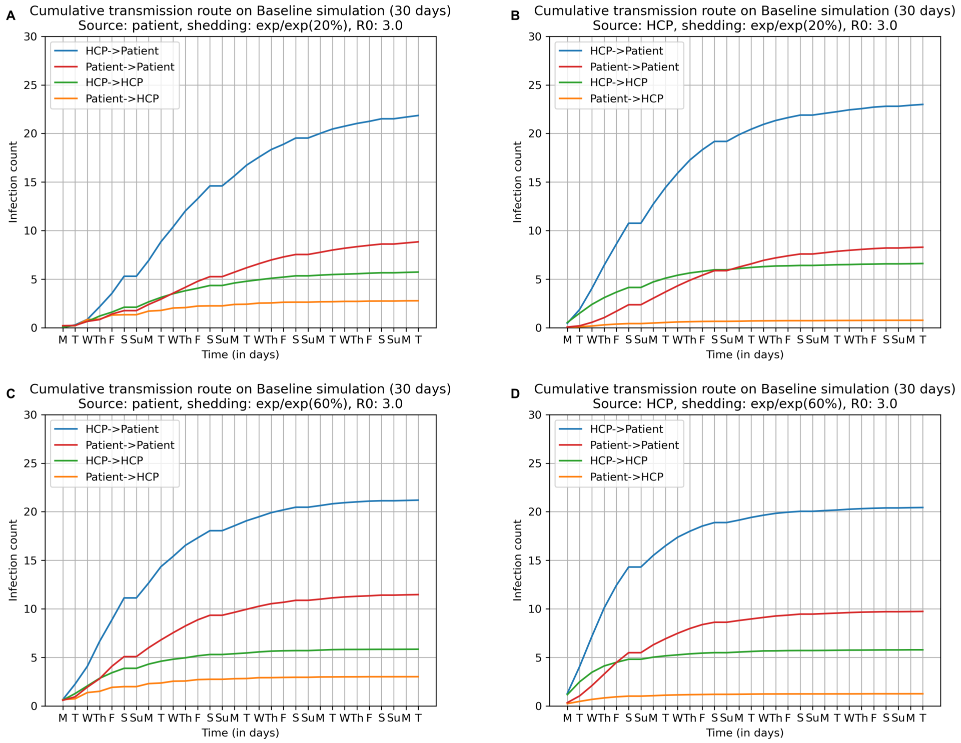

Fig AF. Cumulative distributions of transmission events over 30 days in Baseline simulation on Day 7 and $R_0 = 3.0$. (A) the *exp/exp (20%)* shedding model on Scenario 1. (B) the *exp/exp (20%)* shedding model on Scenario 2. (C) the *exp/exp (60%)* shedding model on Scenario 1. (D) the *exp/exp (60%)* shedding model on Scenario 2.

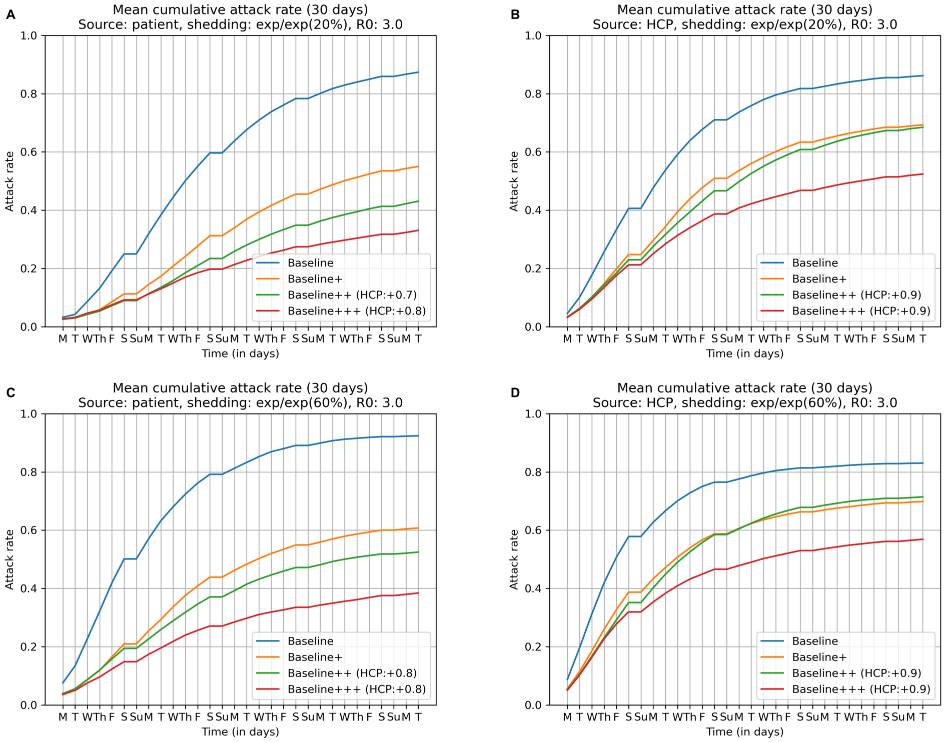

Fig AG. Attack rates for combinations of inexpensive NPIs on Day 7, $R_0 = 3.0$. (A) the *exp/exp (20%)* shedding model on Scenario 1. (B) the *exp/exp (20%)* shedding model on Scenario 2. (C) the *exp/exp (60%)* shedding model on Scenario 1. (D) the *exp/exp (60%)* shedding model on Scenario 2.

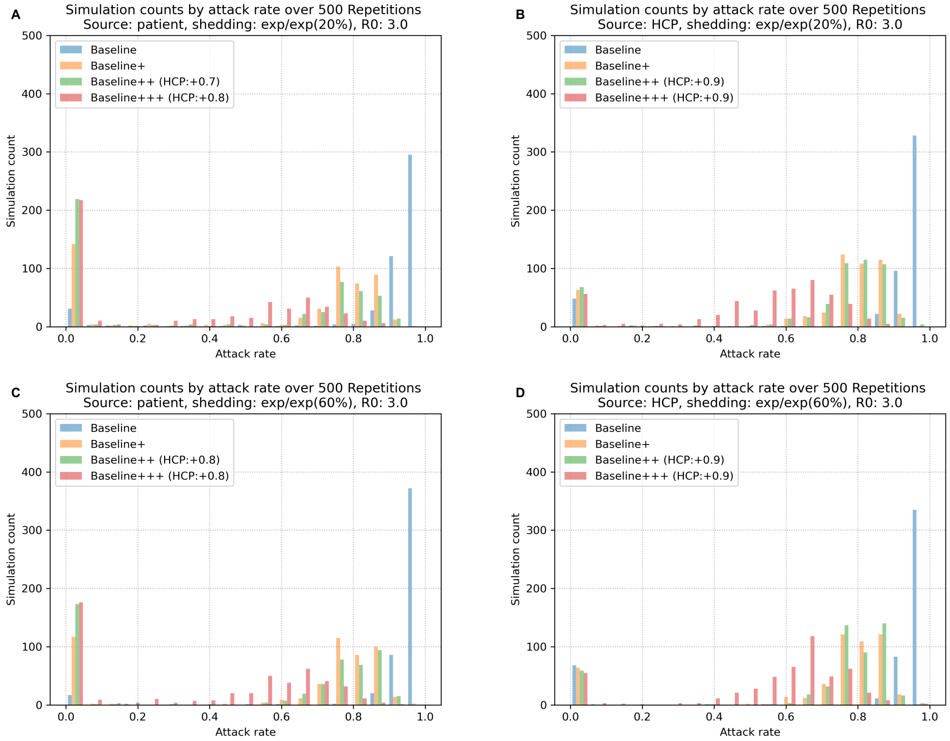

Fig AH. Frequency of replicates as a function of attack rates for combinations of inexpensive NPIs on Day 7, $R_0 = 3.0$. (A) the *exp/exp (20%)* shedding model on Scenario 1. (B) the *exp/exp (20%)* shedding model on Scenario 2. (C) the *exp/exp (60%)* shedding model on Scenario 1. (D) the *exp/exp (60%)* shedding model on Scenario 2.

1.4.4 Day 8

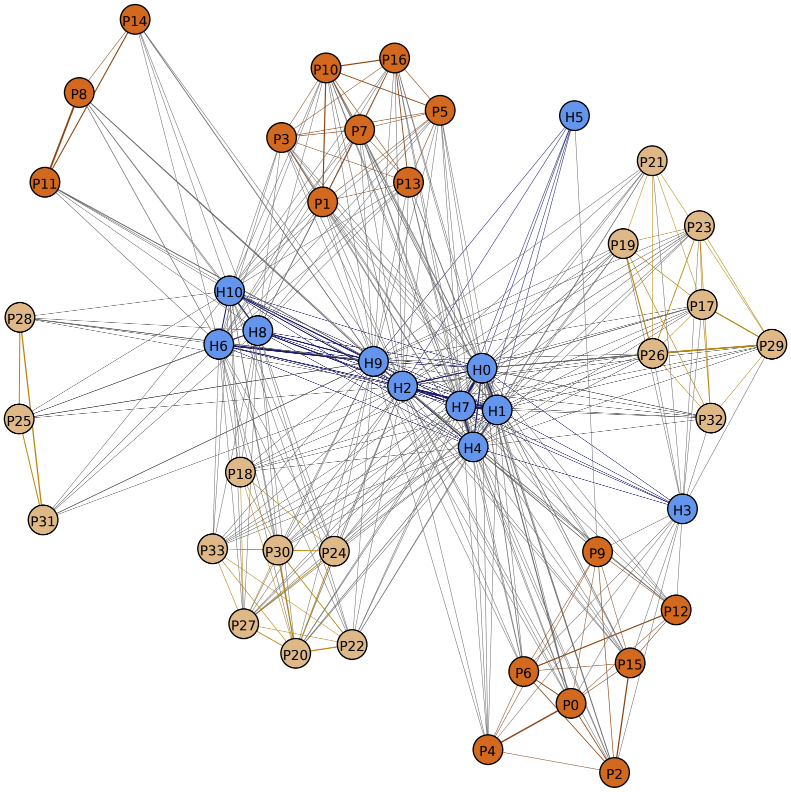

Fig AI. Contact network G on Day 8. We gathered ten days of HCP movement and interaction data where six days had 14.5-15 hours of observation (Day 2, Day 6, Day 7, Day 8, Day 9, and Day 10). HCP nodes, MWF patient nodes, and TThS patient nodes are depicted in blue, chocolate, and burlywood colors, respectively. We observe a total population of 45 agents (11 HCPs and 34 patients).

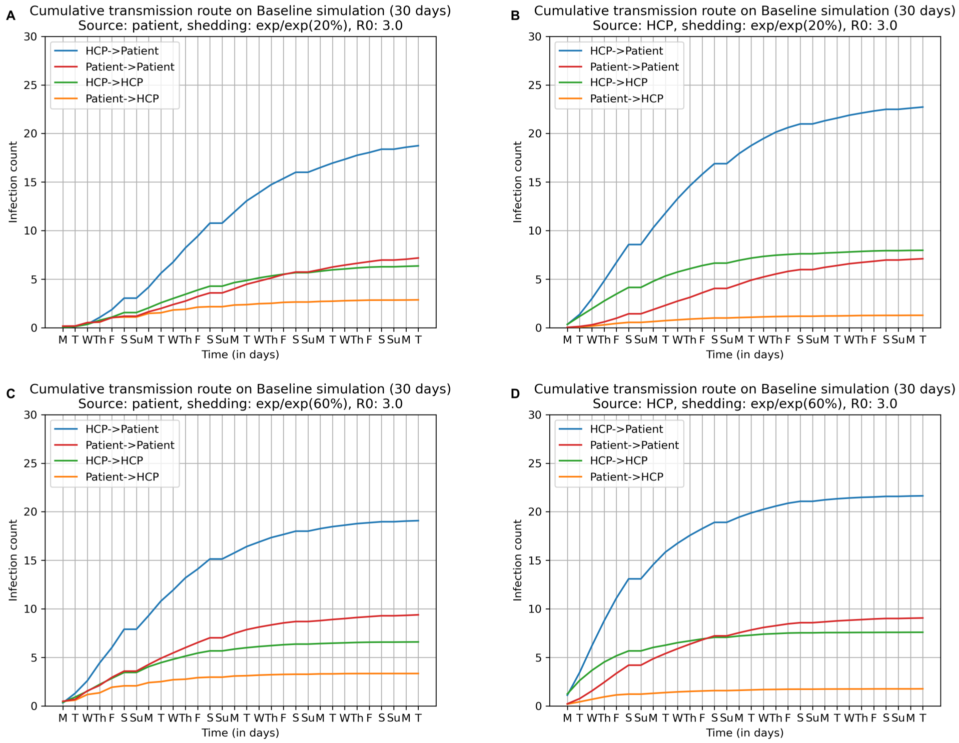

Fig AJ. Cumulative distributions of transmission events over 30 days in Baseline simulation on Day 8 and $R_0 = 3.0$. (A) the *exp/exp (20%)* shedding model on Scenario 1. (B) the *exp/exp (20%)* shedding model on Scenario 2. (C) the *exp/exp (60%)* shedding model on Scenario 1. (D) the *exp/exp (60%)* shedding model on Scenario 2.

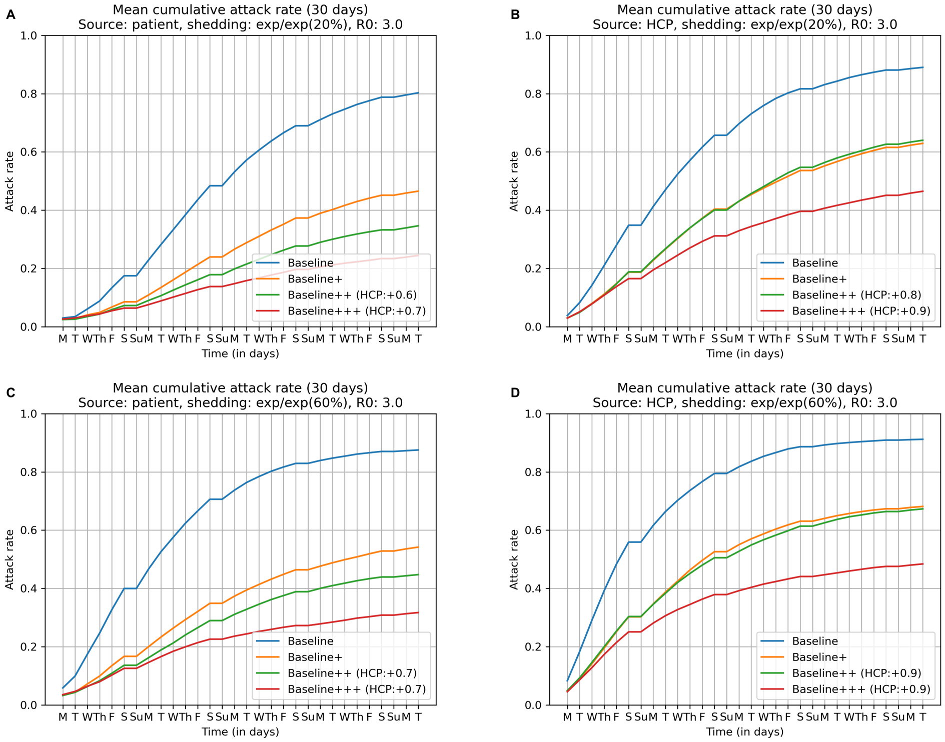

Fig AK. Attack rates for combinations of inexpensive NPIs on Day 8, $R_0 = 3.0$. (A) the *exp/exp (20%)* shedding model on Scenario 1. (B) the *exp/exp (20%)* shedding model on Scenario 2. (C) the *exp/exp (60%)* shedding model on Scenario 1. (D) the *exp/exp (60%)* shedding model on Scenario 2.

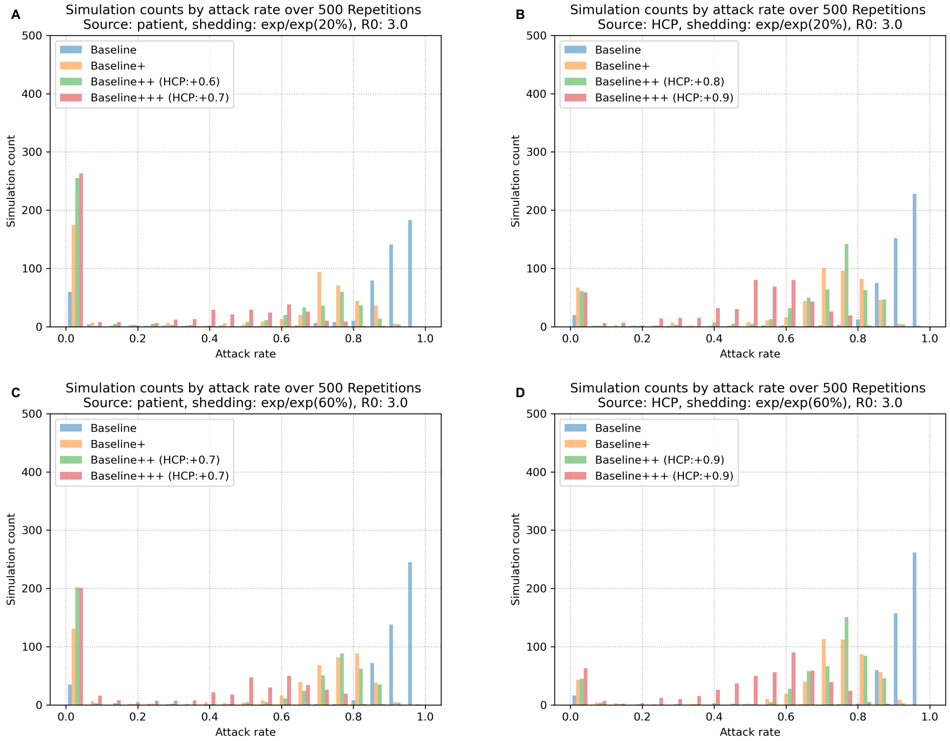

Fig AL. Frequency of replicates as a function of attack rates for combinations of inexpensive NPIs on Day 8, $R_0 = 3.0$. (A) the *exp/exp (20%)* shedding model on Scenario 1. (B) the *exp/exp (20%)* shedding model on Scenario 2. (C) the *exp/exp (60%)* shedding model on Scenario 1. (D) the *exp/exp (60%)* shedding model on Scenario 2.

1.4.5 Day 9

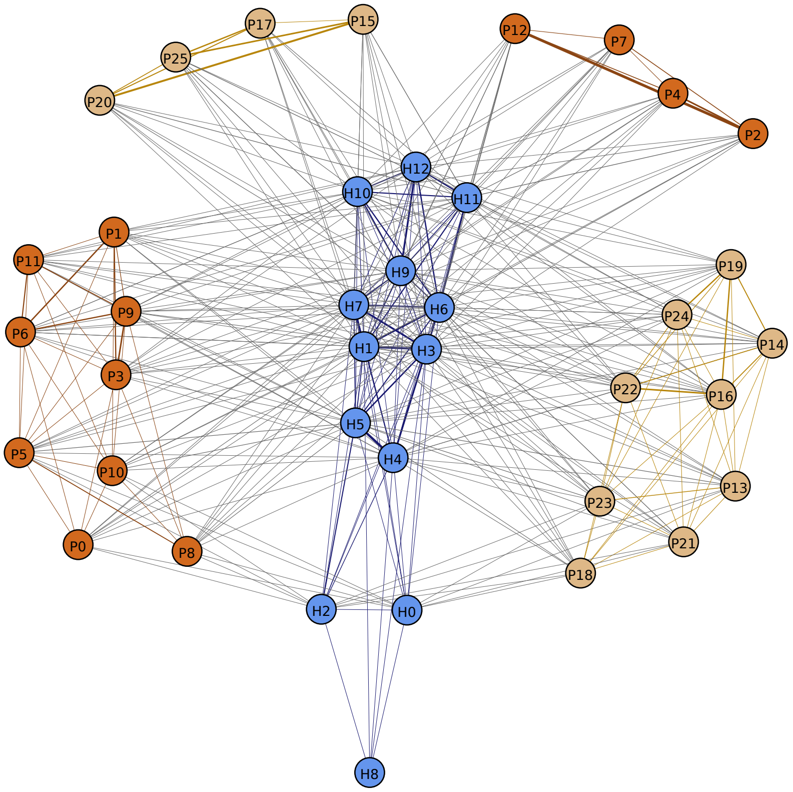

Fig AM. Contact network G **on Day 9.** We gathered ten days of HCP movement and interaction data where six days had 14.5-15 hours of observation (Day 2, Day 6, Day 7, Day 8, Day 9, and Day 10). HCP nodes, MWF patient nodes, and TThS patient nodes are depicted in blue, chocolate, and burlywood colors, respectively. We observe a total population of 39 agents (13 HCPs and 26 patients).

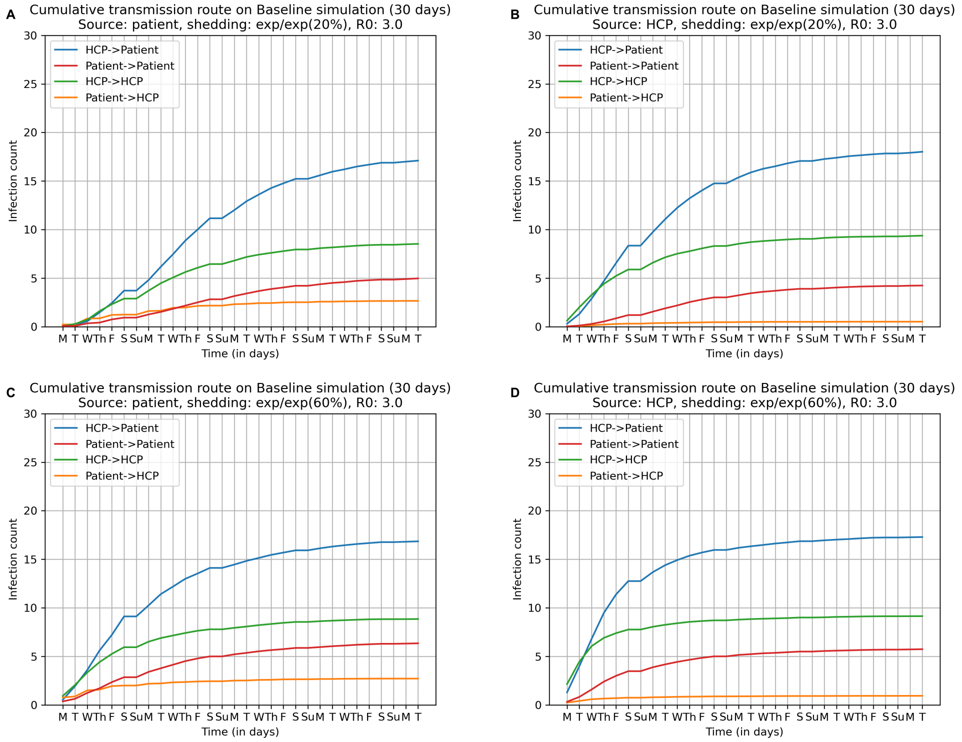

Fig AN. Cumulative distributions of transmission events over 30 days in Baseline simulation on Day 9 and $R_0 = 3.0$. (A) the *exp/exp (20%)* shedding model on Scenario 1. (B) the *exp/exp (20%)* shedding model on Scenario 2. (C) the *exp/exp (60%)* shedding model on Scenario 1. (D) the *exp/exp (60%)* shedding model on Scenario 2.

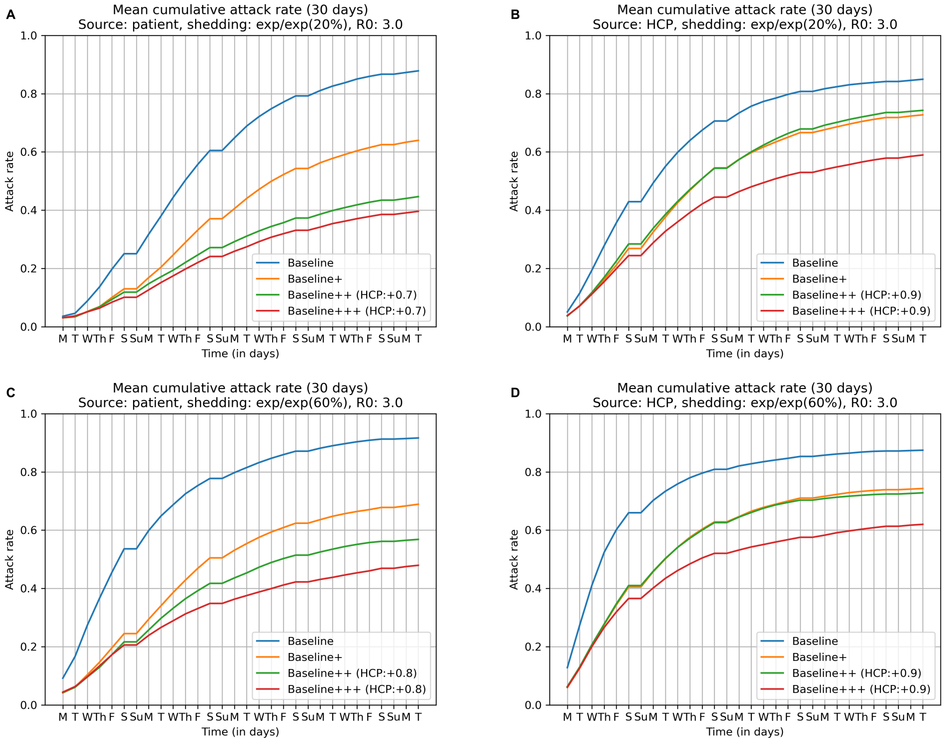

Fig AO. Attack rates for combinations of inexpensive NPIs on Day 9, $R_0 = 3.0$. (A) the *exp/exp (20%)* shedding model on Scenario 1. (B) the *exp/exp (20%)* shedding model on Scenario 2. (C) the *exp/exp (60%)* shedding model on Scenario 1. (D) the *exp/exp (60%)* shedding model on Scenario 2.

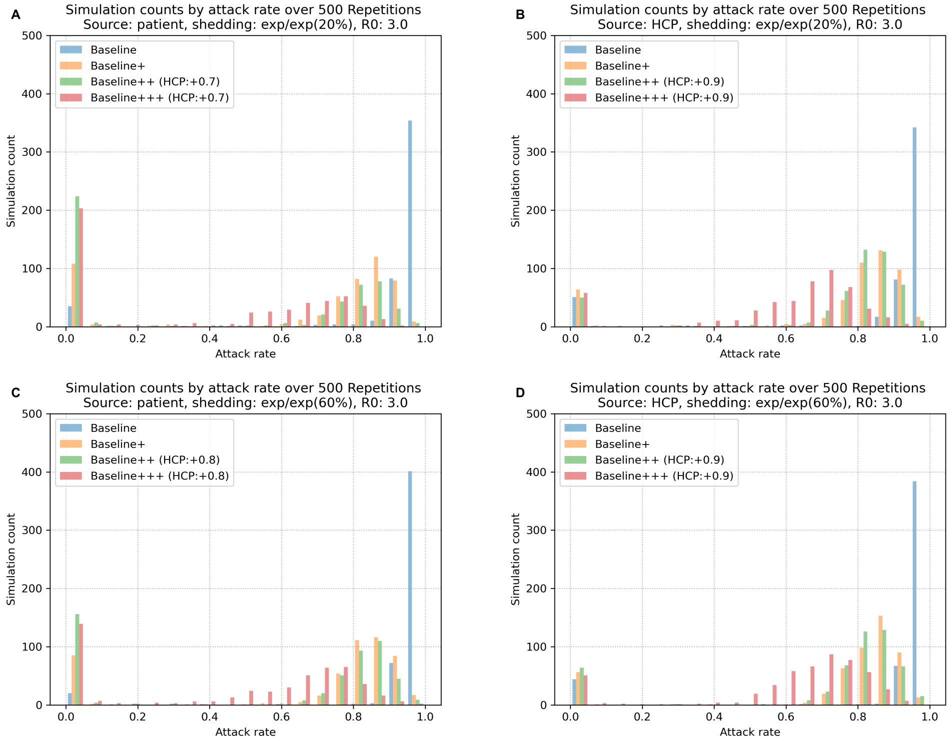

Fig AP. Frequency of replicates as a function of attack rates for combinations of inexpensive NPIs on Day 9, $R_0 = 3.0$. (A) the *exp/exp (20%)* shedding model on Scenario 1. (B) the *exp/exp (20%)* shedding model on Scenario 2. (C) the *exp/exp (60%)* shedding model on Scenario 1. (D) the *exp/exp (60%)* shedding model on Scenario 2.
